# Variation of Site-Specific Glycosylation Profiles of Recombinant Influenza Glycoproteins

**DOI:** 10.1016/j.mcpro.2024.100827

**Published:** 2024-08-10

**Authors:** Zachary C. Goecker, Meghan C. Burke, Concepcion A. Remoroza, Yi Liu, Yuri A. Mirokhin, Sergey L. Sheetlin, Dmitrii V. Tchekhovskoi, Xiaoyu Yang, Stephen E. Stein

**Affiliations:** Mass Spectrometry Data Center, National Institute of Standards and Technology, Gaithersburg, Maryland, USA

**Keywords:** influenza A, glycosylation, hemagglutinin, neuraminidase, site-specific, GADS

## Abstract

This work presents a detailed determination of site-specific N-glycan distributions of the recombinant influenza glycoproteins hemagglutinin (HA) and neuraminidase. Variation in glycosylation among recombinant glycoproteins is not predictable and can depend on details of the biomanufacturing process as well as details of protein structure. In this study, recombinant influenza proteins were analyzed from eight strains of four different suppliers. These include five HA and three neuraminidase proteins, each produced from a HEK293 cell line. Digestion was conducted using a series of complex multienzymatic methods designed to isolate glycopeptides containing single N-glycosylated sites. Site-specific glycosylation profiles of intact glycopeptides were produced using a recently developed method and comparisons were made using spectral similarity scores. Variation in glycan abundances and distribution was most pronounced between different strains of virus (similarity score = 383 out of 999), whereas digestion replicates and injection replicates showed relatively little variation (similarity score = 957). Notably, glycan distributions for homologous regions of influenza glycoprotein variants showed low variability. Due to the multiple possible sources of variation and inherent analytical difficulties in site-specific glycan determinations, variations were individually examined for multiple factors, including differences in supplier, production batch, protease digestion, and replicate measurement. After comparing all glycosylation distributions, four distinguishable classes could be identified for the majority of sites. Finally, attempts to identify glycosylation distributions on adjacent potential N-glycosylated sites of one HA variant were made. Only the second site (NnST) was found to be occupied using two rarely used proteases in proteomics, subtilisin and esperase, both of which did selectively cleave these adjacent sites.

## Highlights

• Variation of site-specific glycosylation measurements is not well understood.
• Glycopeptide abundance distribution spectra can quantitatively describe variation.
• Factors that produce the least variation include replicates and batches.
• Factors that produce the most variation include strain and meta-heterogeneity.
• Conserved regions of hemagglutinin are similar in glycosylation distribution.

## Graphical Abstract

**Figure:**
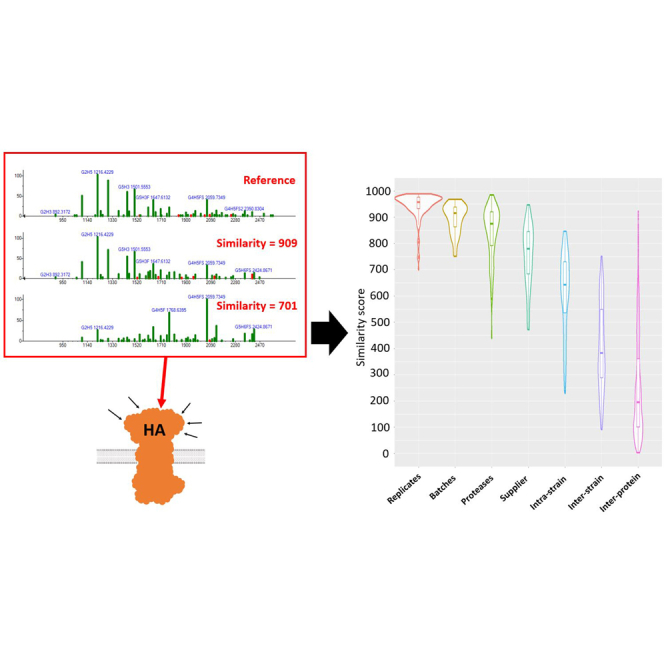

## In Brief

Site-specific glycosylation analyses use measurements from high-resolution mass spectrometry and are often reported with no knowledge of their reproducibility or causes of variation. This study demonstrates how reproducible these measurements are and by what degree certain factors contribute to variation. Factors such as replicates and production batches result in very little variation, whereas viral strain and meta-heterogeneity contribute greatly to variation. Results are also reported here which compare conserved and nonconserved regions of influenza glycoproteins and different unrelated glycoproteins.

Influenza virus is an enveloped pathogen that contains two surface glycoproteins, hemagglutinin (HA) and neuraminidase (NA), which are responsible for viral entry and exit from host cells in the upper respiratory tract (1, 2, 3). These proteins are the main targets of modern influenza vaccines and antiviral agents. One important biochemical characteristic of these proteins is that they contain multiple N-linked glycosylation sites, whose characterization is needed for a molecular-level understanding of viral immune evasion, structure-function relationships, protein folding, and virus entry/exit mechanisms (4, 5, 6, 7, 8). It is well known that the distribution of glycans is unpredictable and can vary greatly between sites on a protein. Therefore, glycan distribution is one of the most significant uncertainties when considering protein structure (4, 9, 10). Relatively recent developments in mass spectrometry have increased the sensitivity and mass accuracy of these measurements, leading to an increasingly detailed picture of site-specific glycosylation. Nevertheless, significant problems remain due to the low abundance and false positive risk of individual glycopeptides. While site-specific studies have been conducted on several influenza glycoproteins (11, 12, 13, 14, 15, 16, 17, 18, 19), the present work extends these studies with a focus on measurement accuracy and variation in glycosylation determinations from a number of sources using NIST developed software tools such as Make-GADS and MS_Piano described in earlier work (20, 21).

Reliably determining site-specific glycosylation distributions involves a number of difficulties which can lead to significant variability (22, 23, 24). This work uses previously developed methods (20, 21, 25) to measure variability associated with by many discrete factors, including digestion reproducibility, protease selectivity, protein sequence, and supplier. Recombinant proteins are well suited for site-specific studies because they are relatively pure, easily obtained, and have a clear origin. Additionally, recombinant proteins have recently gained interest as an alternative vaccine source (26). Here, five hemagglutinin (HA) recombinant proteins were analyzed from subtypes H1, H3, and H5, while three NA recombinant proteins were analyzed from subtypes N1 and N7. These were chosen as exemplars due to both previous study and the extensive lineage of related strains. While these strains are generally similar in sequence and structure, they do have differing primary sequences and therefore the number of glycosylation sites also differs (supplemental Figs. S1 and S2). However, even equivalent glycosylation sites from different strains of HA and NA exhibit quite different distributions of attached glycans (12, 13, 18, 19).

**Fig. 1:**
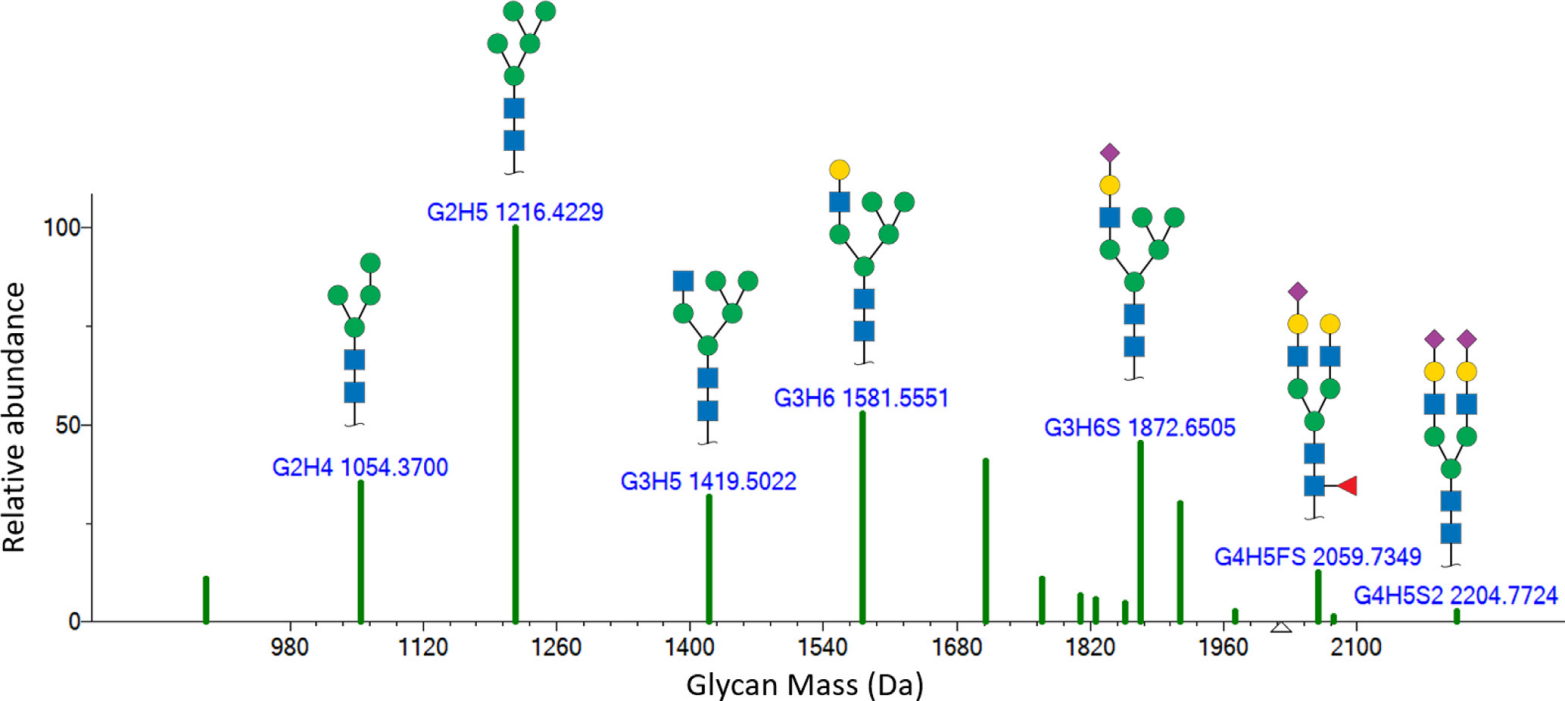
**Illustration of glycopeptide abundance distribution spectrum.** A GADS spectrum is a pseudospectrum representing the glycosylation profiles of a single glycosylation site for a single peptide sequence and charge state with abundances reflecting relative MS^1^ abundances of the underlying glycopeptides. G represents HexNac (N-acetylhexosamine), H represents hexose, F represents fucose (deoxyhexose), and S represents sialic acid (Neu5Ac, N-acetylneuraminic acid). This figure is for illustrative purposes only.

Though focused on influenza glycoproteins, a key goal of this work is to quantitatively examine the reproducibility of site-specific measurements. To facilitate this comparison of site-specific glycosylation patterns, our group has developed an analysis pipeline leading to glycopeptide abundance distribution spectra (GADS) (21). GADS are pseudospectra containing these distributions with the *x*-axis consisting of glycan masses and *y*-axis as corresponding glycopeptide MS^1^ abundances for identified glycans bound to the same peptide ion or ions (illustrated in Fig. 1). This spectral visualization of glycan distributions enables use of available spectral similarity tools and concepts allowing straightforward comparisons of diverse glycosylation distributions. As demonstrated in earlier work, these glycosylation distributions may be readily stored, examined and compared as spectra in searchable spectral libraries (21). Moreover, recent studies have demonstrated a quantitative relationship between relative glycopeptide abundances as presented in site-specific GADS and relative concentrations of their glycans in the protein (27). Other objectives of this work are to expand the use of site-specific glycosylation analysis methods to a wider variety of proteins and to establish a quantitative point of comparison in terms of variation and reproducibility for future studies of N-glycosylation. Finally, we report results for two proteases not commonly used in proteomics that are found to cleave adjacent potential N-glycosylation sites.

## Experimental Procedures

### Experimental Design and Statistical Rationale

Recombinant HA and NA were obtained from four commercial suppliers (Table 1). Six protease combinations were used to isolate individual N-glycosylation sites from each protein, which are characterized by asparagine residues in a NX[S/T] arrangement, where X is not P. Noncanonical glycosylation sequons such as NXC were not included in this work due to the infrequency of their observations. Additionally, four nonspecific proteases in combination with and without trypsin were used in an effort to isolate adjacent glycosylation sites.

**Table 1:** Recombinant proteins analyzed for site-specific glycosylation

Abbreviation	Protein	Strain	Subtype	Supplier	Glycosylation sites (adjacent pairs/total)	Protein mass (kDa) unglycosylated
HA-CA09	HA	A/California/04/2009	H1N1	1	1/8	63
HA-NC99	HA	A/New Caledonia/20/1999	H1N1	2	1/10	63
HA-JP57	HA	A/Japan/305/1957	H2N2	1	2/8	63
HA-HK14	HA	A/Hong Kong/485197/2014	H3N2	3	0/13	64
HA-HK97	HA	A/Hong Kong/483/1997	H5N1	2, 3, 4	1/8	64
NA-AZ08	NA	A/Arizona/13/2008	H1N1	2	0/9	52
NA-TH04	NA	A/Thailand/1 (KAN-1)/2004	H5N1	2, 3, 4	0/3	49
NA-NL03	NA	A/Netherlands/219/2003	H7N7	1	2/11	52

Abbreviations: HA, hemagglutinin; NA, neuraminidase.

For GADS comparisons in this work, multiple replicate types were performed. These include six repeat digestions of HA-HK97 and NA-TH04 from supplier 4 and a total of nine repeat injections. This resulted in 142 GADS comparisons. Number of samples analyzed and number of GADS comparisons made for all other factors are as follows: (1) batch, 17 and 56; (2) protease, 35 and 111; (3) supplier, 33 and 100; (4) intrastrain, 8 and 281; (5) intraprotein, 48 and 193; and (6) interprotein, 88 and 10,077. See supplemental Table S1 for additional information about samples and types of replicates analyzed. Altogether, 128 LC-MS/MS runs were injected. NIST-developed software was used to create GADS, (21) which are used to compare variations between the factors listed above. Traditional pattern matching similarity scoring (dot product) was used to assess GADS variations (28).

### Influenza Strains and Glycoproteins

Recombinant HA and NA were selected as glycoprotein candidates in this study. Proteins were obtained from four suppliers including Sino Biological, BioVision, US Biological, and Creative Biomart which are deidentified in this work as suppliers 1 to 4 (Table 1 and supplemental Table S2). These glycoproteins and strains were selected based on their use in relevant literature (12, 13, 15, 17, 18), availability among different suppliers, large number of glycosylation sites, and presence of directly adjacent glycosylation sites (*e.g.*, NNST). All recombinant glycoproteins acquired were expressed in HEK293 cell lines and are full-length proteins with variably trimmed C-terminus and N-terminus and addition of a histidine tag at the C-terminus for purification. Glycoproteins analyzed here include HA from strain A/California/04/2009 (Creative Biomart HA-283V), HA from strain A/New Caledonia/20/1999 (Sino Biological 11683-V08H), HA from strain A/Japan/305/1957 (Creative Biomart HA-299V), HA from strain A/Hong Kong/485197/2014 (BioVision P1236-50), HA from strain A/Hong Kong/483/1997 (Sino Biological 11689-V08H, BioVision P1005-20, US Biological 519006), NA from strain A/Arizona/13/2008 (Sino Biological 40734-V07H), NA from strain A/Thailand/1(KAN-1)/2004 (Sino Biological 40064-V07H, BioVision 7508-20, US Biological 519024), and NA from strain A/Netherlands/219/2003 (Creative Biomart NA-716V).

### Protein Digestion and Desalting

The method for glycoprotein digestion was adapted from the RapiGest user guide (29). In summary, 5 μg of protein was added to 25 μl of 0.1% RapiGest (w/v) + 50 mmol/L ammonium bicarbonate in a 1.5 ml Eppendorf tube with purified water from a Milli-Q system. DTT was then added to reach a final concentration of 20 mmol/L. The solution was left to incubate at 60 °C for 1 h in an Eppendorf thermomixer at 450 rpm. After reduction, the reaction tube was cooled to room temperature and iodoacetamide added to reach a concentration of 55 mmol/L. The tube was again left to incubate at room temperature for 45 min in an Eppendorf thermomixer at 450 rpm with a covered cap. After cysteine alkylation, DTT was added to achieve a concentration of 60 mmol/L to quench excess iodoacetamide. The sample was then diluted to ≈250 μl with 50 mmol/L ammonium bicarbonate to make the RapiGest final concentration ≤0.01%.

Using HA-HK97 and NA-TH04 from three suppliers, experimental reproducibility was examined in two ways: (1) reinjections of the same digest (injection replicates) and (2) repeat digestions of the same starting protein material (digestion replicates). The five principal proteases were purchased from two suppliers. These include sequencing grade trypsin (Promega V511A), Glu-C (Promega V165A), Lys-C (Promega V167A), chymotrypsin (Promega V106A), and alpha-lytic WT protease (Sigma A6362). Six protease combinations were used for digestion of the recombinant glycoproteins: (1) trypsin with Glu-C, (2) trypsin with Lys-C, (3) trypsin with chymotrypsin, (4) chymotrypsin with Glu-C, (5) chymotrypsin alone, and (6) alpha-lytic WT protease alone. Optimization of digestion conditions is described elsewhere (30). Digestion conditions varied based on the proteases used. For digestion using trypsin/Glu-C and trypsin/Lys-C, the proteases were added in parallel (each at 1:50 mg enzyme: mg substrate) and shaken for 18 h in a thermomixer at 37 °C and 450 rpm. For digestion using chymotrypsin, trypsin/chymotrypsin, and Glu-C/chymotryspin, the proteases were added in parallel (1:50 wt/wt each) and shaken for 18 h in a thermomixer at room temperature and 450 rpm. For digestion using alpha-lytic protease, the first addition of the protease was added (1:100 wt/wt) and shaken for 1 h in a thermomixer at 37 °C and 450 rpm and then a second addition was made under the same conditions. In a limited series of studies, subtilisin (Sigma P5380), neutrase (Sigma P1236), esperase (Sigma P5860), and savinase (Sigma P3111) additions were conducted at 1:50 wt/wt concentration and digested for 30 min at 56 °C. Different times were used for these proteases (20 min–60 min) along with codigestion with trypsin. Protease activity was stopped by the addition of HPLC grade TFA to reach a final concentration of 0.2% (v/v).

Glycoprotein digests were desalted using MonoSpin C18 SPE spin centrifuge columns (GL sciences). Each column was activated twice with 200 μl of HPLC grade acetonitrile (ACN), followed by centrifugation for 1 min at 2300 relative centrifugal force (rcf). Each column was then equilibrated twice with 200 μl of 0.1% TFA, followed by the same centrifugal conditions. The flow through was then discarded and the sample digests placed in the MonoSpin C18 columns and centrifuged for 2 min at 2300 rcf. Bound peptides were then washed twice using 200 μl of 0.1% (v/v) TFA followed by centrifugation for 1 min at 2300 rcf. The flow through was discarded. Peptides were then eluted from the column using two rounds of 100 μl 60% ACN/0.5% formic acid (v/v) with the same centrifugal conditions and two rounds of 100 μl 80% ACN/0.5% formic acid (v/v) with the same centrifugal conditions. Eluted peptides were then dried down using a Genevac EZ-2 plus Speedvac (SP Scientific) at HPLC mode at 40 °C for about 1 h or until dry. Samples were then reconstituted with 28.5 μl of 2% ACN/0.1% formic acid (v/v) to be at a concentration of 175 ng/μl.

### Site-Specific Glycopeptide Analysis

Samples were analyzed using an Orbitrap Fusion Lumos Tribrid mass spectrometer with attached nanospray Flex ion source and in line with an Ultimate 3000 nLC HPLC (Thermo Fisher Scientific). Most samples were loaded at 350 ng of digested peptide material on an Acclaim PepMap RSLC 75 μm × 25 cm reverse phase column (Thermo Fisher Scientific) and separated by either a 230 min gradient or 180 min gradient at 300 nl/min flow rate. Some samples were loaded at the same concentration on an Acclaim PepMap RSLC 75 μm × 15 cm reverse phase column (Thermo Fisher Scientific) and separated on a 150 min gradient at 300 nl/min flow rate. These changes in gradient were made due to changing laboratory conditions and did not have a noticeable effect on results. Distinctions between these are given in the raw file name, designated 230 min, 150 min, or 180 min. Each run began at 2% ACN. For the 230 min run, the initial gradient was held for 25 min, increased to 32% ACN at 142 min, increased to 98% ACN at 165 min and held 10 min, decreased to 2% ACN at 180 min and held until the end of the run. For the 180 min run, the gradient increased to 32% ACN at 117 min, decreased to 0% ACN at 120 min, increased to 80% at 134 min, increased to 98% at 140 min and held for 10 min, decreased to 2% ACN at 155 min, and held until the end of the run. For the 150 min run, the initial gradient increased to 35% ACN at 100 min, increased to 98% ACN at 105 min and for 15 min, decreased to 2% ACN at 125 min, and held until the end of the run.

Full spectra mass spectrometric acquisition was performed with a scan range of 380 *m/z* to 2000 *m/z* and detection in the orbitrap at a resolution of 120,000. The maximum ion injection time was 50 ms with automatic gain control target of 400,000 and a radio frequency lens value of 40%. Filters include charge states 2 to 8, minimum intensity threshold of 5 × 10^4^, maximum intensity threshold of 1 × 10^20^, and a dynamic exclusion of 15 s, and a total cycle time of 5 s. Precursor priority for the data-dependent acquisition was filtered according to the highest charge state and lowest *m/z*. Quadrupole isolation mode was used with an isolation window of 2 *m/z* for tandem mass spectra acquisition. Two types of MS^2^ spectra were acquired in the orbitrap: one resulting from stepped fragmentation and the other one resulting from contingent ion trap fragmentation. Both methods used a maximum injection time of 60 ms, an AGC target of 50,000, and a resolution of 30,000 in the orbitrap. Stepped fragmentation used beam-type collision-cell spectra from higher energy collisional dissociation (HCD) at 15%, 25%, and 35% normalized collision energy as optimized in previous work (4). Ion trap fragmentation was contingent upon the detection of HexNAc oxonium ion (*m/z* = 204.087) in the HCD spectrum. Ion trap fragmentation was done at a collision energy of 30% and 10 ms of activation time.

### Data Processing

Glycopeptide identifications and abundances were primarily derived from Byonic software output (version 3.10.10, https://proteinmetrics.com/byonic/). FASTA sequences used for searching only included a single protein, corresponding to the protein and strain of interest for each run. Search parameters included three maximum missed cleavages, with six maximum missed cleavages for alpha-lytic protease, a precursor mass tolerance of 5 ppm, and fragment mass error of 20 ppm. cysteine carbamidomethylation (+57.02) was set as a fixed modification and variable modifications included methionine oxidation (+15.99) and glutamine N-terminal ammonia loss (−17.03). The glycan database used for searching consists of 444 human N-glycans and is composed of glycans found in mammalian, human, and sulfation databases (4). Output files used for data analysis include the xlsx and csv files from Byonic software. Within the data processing pipeline, false discovery rate was not used for validation of glycopeptide MS^2^ spectra since it does not consider multiple factors used in the present validation scheme such as retention time, presence of oxonium ions, and consistency of HCD and ion trap spectra. For the nonstandard proteases subtilisin and esperase, MSfragger-glyco (31) in nonselective mode was used for GADS determination (https://msfragger.nesvilab.org/). In our recent work (27), this method was found to produce results very similar to Byonic.

Annotated tandem glycopeptide libraries were created using a suite of in-house software which includes MS_Piano for the annotation of tandem spectra for N-glycopeptides (https://chemdata.nist.gov/dokuwiki/doku.php?id=peptidew:ms_piano) (20). MS_Piano annotates both peptides and intact N-glycopeptides by sequence, charge state, and modification. Spectra shown in the paper employed this annotation. The suite of software used for tandem library creation begins with output data from Byonic which tentatively identifies glycopeptides and provides extracted ion chromatogram (XIC) information from MS^1^ data. Identified glycopeptides are then subject to further validation based on contingent ion-trap collision-induced dissociation fragmentation spectra, retention time, Y ion presence, purity, XIC overlap, and number of identified HCD spectra found. The annotated and validated glycopeptide spectra are then converted into NIST MS library format. Two different, well-identified glycopeptides were required for GADS generation. Resulting GADS libraries can be visualized, compared, and searched within the NIST MS Search software (https://chemdata.nist.gov/dokuwiki/doku.php?id=chemdata:downloads:start#nist_ms_search_program).

GADS were created for each digest as a means of storing glycosylation distributions. Each GADS represents many glycoforms for one peptide sequence from one sample injection into the mass spectrometer. Briefly, these pseudospectra were created from MS^2^ glycopeptide spectra with glycan mass as the *x*-axis and their MS^1^ XIC abundances on the *y*-axis. Each GADS peak represents a glycan with monosaccharides represented as follows: G represents HexNac (N-acetylhexosamine), H represents hexose, F represents fucose (deoxyhexose), S represents sialic acid (N-acetylneuraminic acid), and Po represents phosphorylation (21). No sulfation (So) was confidently detected. Red peaks represent less confident glycopeptide identifications that may have several origins, including inconsistent retention time, lack of a Y1 ion, lack of expected oxonium ions, and other factors. See Supplemental Information Pages S7–S8 for further details. GADS represent all glycopeptides from a single sequence and charge state for a single run, enabling direct comparison between glycan distributions and repeat runs for a given sequence. As discussed previously, multiple charge state GADS were constructed when or more individual charge state GADS were generated for a single sequence (21). In-house software was used to create GADS pseudospectra from Byologic and MSfragger output and from validation information from the in-house tandem mass spectral library creation software.

### Data Metrics and Statistical Analysis

Comparisons of glycosylation distributions was conducted using a cosine-like score, long used in NIST library searching, referred to here to as a similarity score or dot product as described by Stein *et al.* (28). This enabled GADS to be sorted according to their similarity. Instead of the mass and abundance axes used in a conventional mass spectrum, glycan masses and MS^1^-derived glycopeptide abundances derived from multiple glycopeptides were used to construct a GADS (21). While GADS that combined multiple charge states were generated, all comparisons in this work were made from single charge state glycopeptides. If a peptide or glycosylation site was not detected in one sample and was detected in the other, no comparisons were made. Variation among these similarity scores, attributed to glycosylation differences, was represented by box and whisker plots and violin plots. Rstudio was used to produce hierarchical clustering dendrograms of GADS spectra to find classes of glycan distributions based on pairwise similarity scores (https://www.R-project.org/).

## Results

**Fig. 2:**
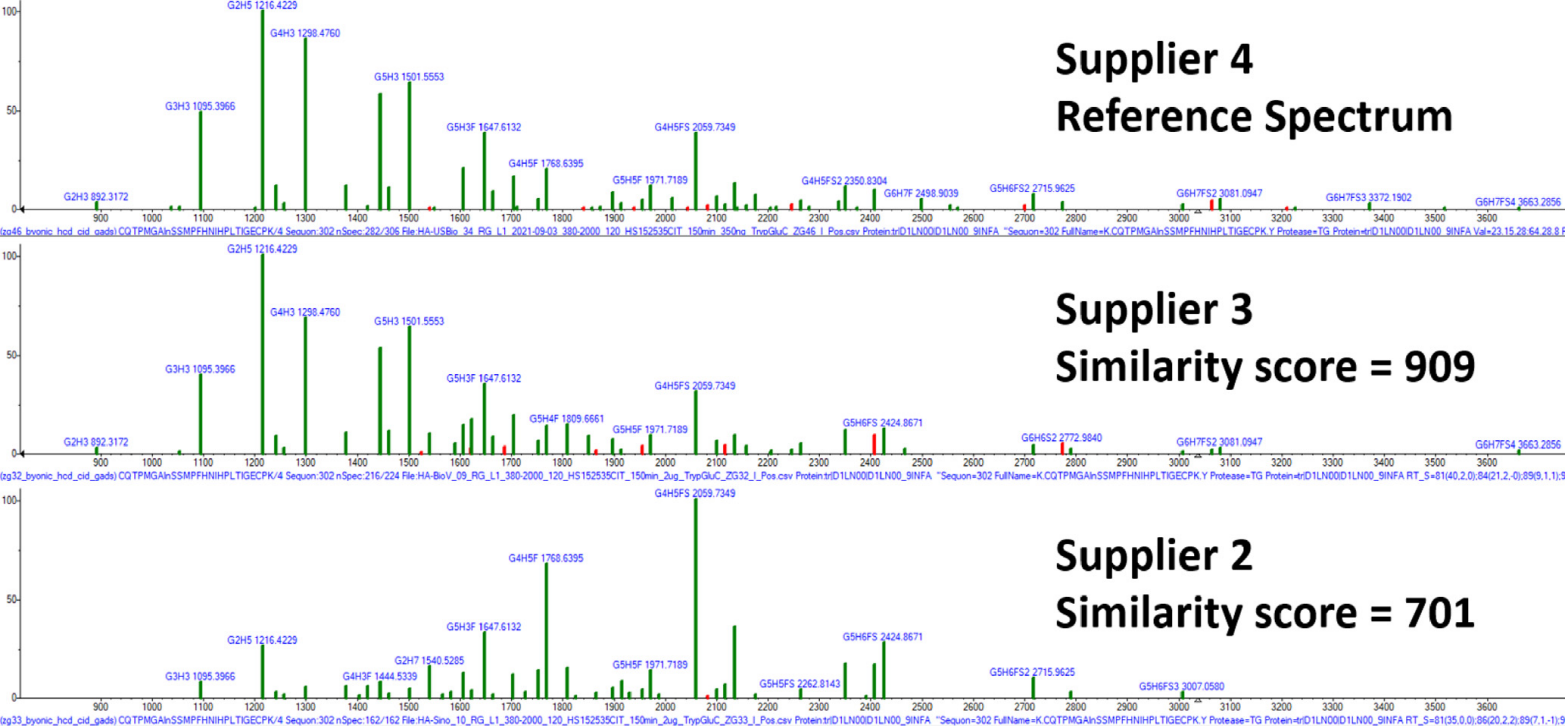
**Example comparison of GADS between three suppliers.** Glycan distributions compared for recombinant hemagglutinin for strain HA-HK97 from suppliers 2 and 3 to supplier 4 at glycosylated position 302 for the peptide sequence CQTPMGAInSSMPFHNIHPLTIGECPK at charge state +4 (“n” denotes glycosylated asparagine). All distributions are the result of digestion using trypsin + Glu-C. GADS, glycopeptide abundance distribution spectra; HA, hemagglutinin.

Results are presented in terms of comparisons of GADS, their similarity scores (dot product), and by specific qualitative differences in glycan distributions. Differences arise between replicate measurements of the same sample, different digestions using the same or different protease digestion of the same protein, suppliers of the same protein and protein strains. Figure 2 provides a typical example of such GADS comparisons. In this and most cases, distributions for HA and NA primarily involve core-fucosylated complex glycans. Distributions on HA were commonly composed of the glycans G2H5, G4H3F, G4H4F, G4H5F, G5H4F, and G4H5FS. Another common distribution included these along with higher mass sialylated glycans such as G5H6FS, G5H6FS2, G6H7FS, and G6H7FS2. Some of the most abundant glycans on NA across all strains include G4H3F, G4H5F, and G5H4F2. For more information on these and other site-specific distributions, see Supplemental Document S1. All distributions measured in this work may be viewed using GADS software provided at Chemdata.nist.gov (https://chemdata.nist.gov/dokuwiki/doku.php?id=peptidew:influenza_a_glycoproteins).

### Overall Variation

**Fig. 3:**
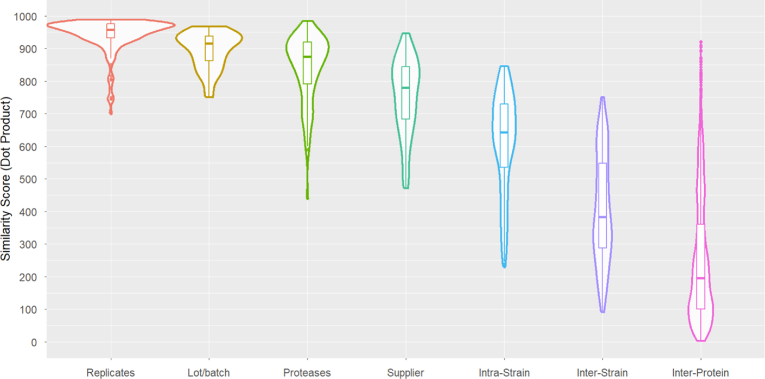
**Violin plot summarizing glycosylation comparisons.** Similarity scores between GADS for replicates (injection and digestion replicates combined), supplier batches, protease combinations, suppliers, intrastrain glycosylation sites, interstrain glycosylation sites, and interprotein GADS similarity between unrelated proteins. The *y*-axis represents dot product similarity. *Box and whiskers markers plots* are included within each violin to show quartiles of the distribution. For replicate, batch, and supplier comparisons, compared GADS had identical peptide sequences and charge states. GADS, glycopeptide abundance distribution spectra.

A number of site-specific glycosylation studies of influenza proteins have recently been reported (11, 12, 13, 14, 15, 16). However, these comparisons typically combine distributions into broad classes such as high-mannose, complex, and hybrid-type glycans. Others report site-specific values in tables or XICs, making it inconvenient to compare results in detail. The present comparisons provide a more complete chemical description of glycosylation as well as a detailed examination of experimental and biological variability. Figure 3 shows variations arising from multiple sources, illustrating the wide range of similarity scores. They include digestion and injection replicates (median similarity score = 957), batches (916), different proteases for digestion (875), suppliers producing the same protein (781), intrastrain (or within-sequence) variation (643), interstrain variation (383), and interprotein variation (198).

**Fig. 4:**
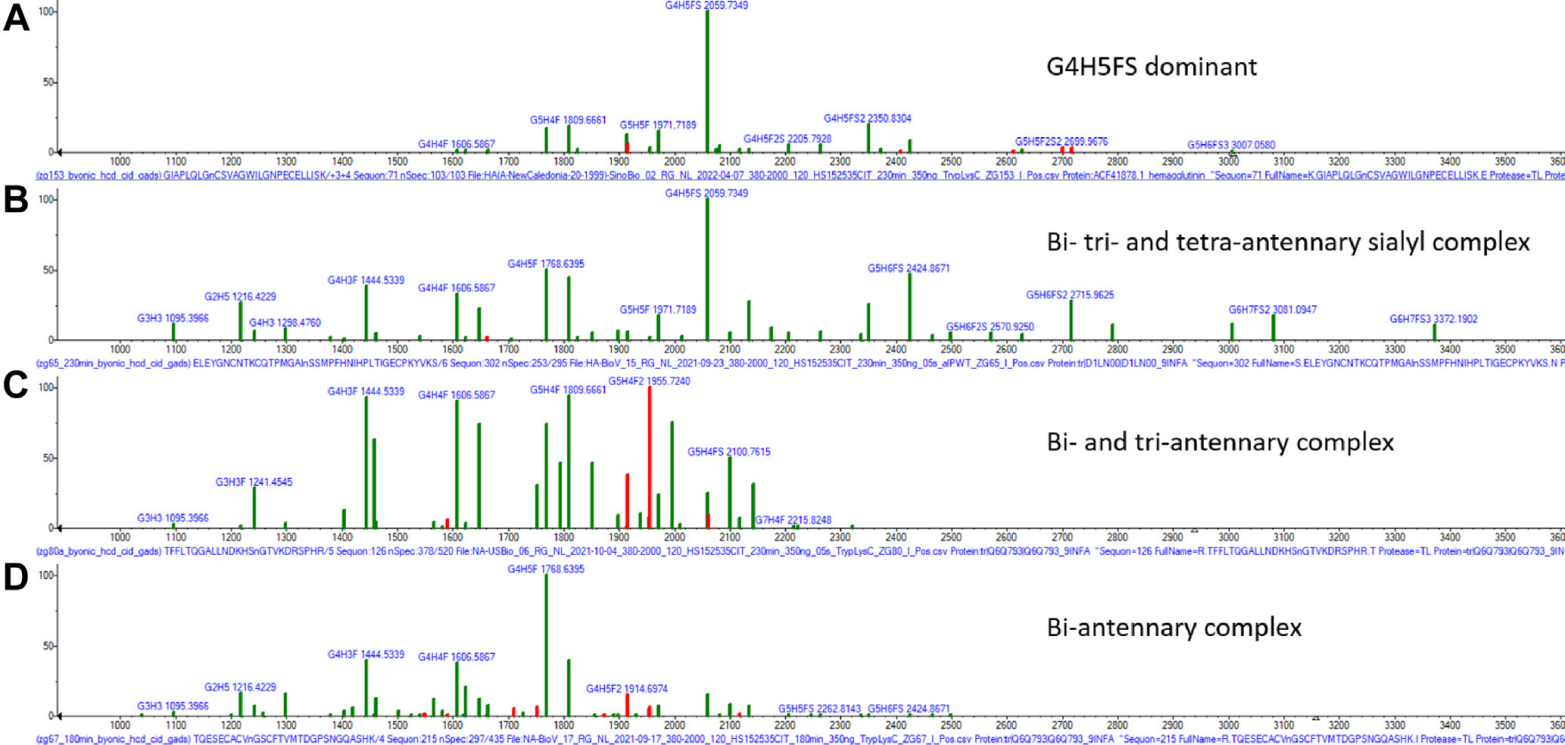
**Common GADS distributions.** Illustrations of four recurring varieties of glycosylation distributions. These were distinguished by intraclass similarity scores greater than 500. *A*, G4H5FS dominant GADS from protein HA-NC99 position 71, (*B*) biantennary, triantennary, and tetra-antennary sialylated complex-type GADS from protein HA-HK97 position 302, (*C*) biantennary and triantennary complex-type GADS from protein NA-TH04 position 126, and (*D*) biantennary complex-type GADS from protein NA-TH04 position 215. *Red peaks* denote less confident identifications. GADS, glycopeptide abundance distribution spectra; HA, hemagglutinin; NA, neuraminidase.

In view of the wide range of glycosylation sites and their sometimes high degree of variability, it is hard to make generalizations about the specific glycans associated with this variability. The libraries of GADS provided with this paper as well as the many GADS in the Supplemental document directly show the origins of this variability. Some glycans are unique to certain suppliers, such as with multiply sialylated complex glycans G6H7FS2 and G6H7FS3 which are unique to supplier 2 and low hexose glycans such as G4H3 and G5H3 are unique to supplier 4. In an effort to find common patterns, simple clustering with a score threshold of 500 indicated that 2/3 of all GADS could be represented as members of four general classes. They are illustrated in Figure 4 and the underlying data shown in detail in supplemental Fig. S3. The average intraclass similarity score is 674, whereas the average interclass similarity score is 340. All four classes predominantly consist of core-fucosylated complex glycans. The first class is primarily composed of sialylated G4H5 complex-type glycans such as G4H5FS (Fig. 4*A*) and is found in both HA and NA. The second class is unique to HA and is characterized by high abundance of biantennary, triantennary, and tetra-antennary complex-type glycans with and without sialylation (Fig. 4*B*). The third class is unique to NA and has a high abundance of mostly nonsialylated biantennary and triantennary complex glycans such as G4H3F and G4H4F (Fig. 4*C*). The final class is unique to NA and has a high abundance of nonsialylated biantennary complex glycans such as G4H4F and G4H5F (Fig. 4*D*).

### Variation Between Replicates and Supplier Batches

Due to their low level of variability, results are combined for injection replicates and digestion replicates in Figure 3 as one category of “replicates.” Five injection duplicates were run for HA-HK97 and four injection duplicates were run for NA-TH04, while three digestion replicates were run for HA-HK97 and three digestion replicates were run for NA-TH04. These repeat measurement types were run independently of each other, resulting in 24 total measurements. Reproducibility among both replicate types is very high, with median GADS similarity scores of 955 and 960 (Table 2), indicating that they are not significant sources of variability (Fig. 3). It is important to note that there are outliers with lower scores. These are invariably due to lower signal strengths and smaller numbers of identified glycopeptide ions. All matching GADS from both proteins had similarity scores above 850 for both replicate types (Table 2). In general, variations diminished with increasing numbers of glycopeptide identifications (supplemental Fig. S4). GADS composed of glycopeptides identified more than 250 times have particularly high degrees of similarity (dot product >950), whereas peptides identified fewer than 50 times almost invariably have a lower degree of similarity (dot product <900). In terms of variation of specific glycans in a distribution between replicates, glycans G4H5FS, G3H3, and G4H4F were the most variable (Table 3 and supplemental Figs. S5–S7).

**Table 2:** Median GADS similarity scores between replicates and batches for HA-HK97 and NA-TH04 from supplier 4

Protein	Site	Injection replicates	Digestion replicates	Different batches
Hemagglutinin	39	963	980	901
	170	967	938	843
	181	926	971	940
	302	962	929	924
	500	936	870	866
Neuraminidase	68	937	971	N/A
	126	973	945	877
	215	955	960	891
Total		955	960	910

Abbreviation: GADS, glycopeptide abundance distribution spectra.

**Table 3:** Glycan comparisons within each factor

Factor	Consistently abundant	Most variable
Replicates	G2H5	G4H5FS
	G4H3	G3H3
	G4H3F	G4H4F
Batches	G2H5	G5H3F
	G4H3	G4H4
	G4H3F	G4H4F
Proteases	G2H5	G2H4
	G4H3	G4H5
	G3H3F	
Supplier	G3H3	G4H3F
	G2H5	G5H6FS
	G5H3F	G5H3
	G4H5FS	
Intrastrain	G2H5	G6H3F
	G4H5F	G5H4FS2
	G4H4F	G3H5FS
	G4H5FS	
Interstrain	G5H4F	G2H9
	G4H5FS	G3H3
	G4H5F	G6H7FS2
	G4H3F	

Differences in glycosylation for two batches of the same protein sequence from a single supplier were determined for samples from suppliers 2, 3, and 4 for proteins NA-TH04 and HA-HK97. Results demonstrate a high median overall similarity score of 910 (Table 2). However, this degree of similarity is lower than deviations from replicates, where the median similarity scores are above 950 (Fig. 3). Only 16% of the scores within the interquartile range (difference between first and third quartiles) for these two groups overlap. This demonstrates that batch-to-batch variation is significantly greater than measurement variation. Here, data suggests that the glycans most inconsistent in abundance between batches include complex glycans such as G5H3F and G4H4F, as well as other low-mass complex glycans such as G4H4 (Table 3 and supplemental Figs. S8 and S9).

### Variation Due to Protease

**Fig. 5:**
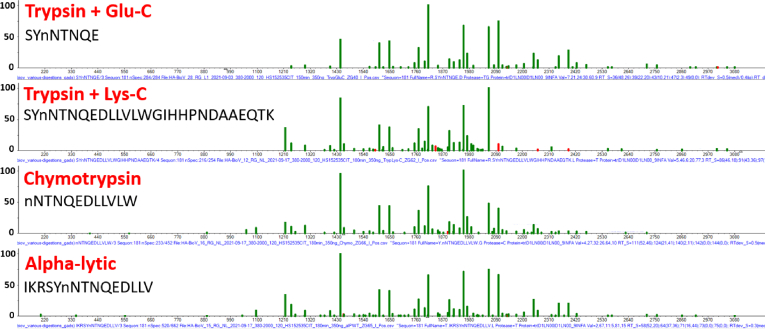
**Different proteases change the peptide landscape around a glycosylation site.** Six protease combinations were used to optimize glycosylation site determination. GADS were compared based on the different peptides produced by the various protease combinations. The *lowercase n* represents the site of glycosylation. GADS compared are from protein HA-HK97 at glycosylation site 181. GADS, glycopeptide abundance distribution spectra; HA, hemagglutinin.

The ability to establish site-specific glycan distributions depends on the ability of a protease or fragmentation method to selectively isolate abundant peptide sequences containing a single glycosylation site (4, 30). Due to differing cleavage selectivity, use of multiple proteases can enhance the overall coverage of glycosylation sites and is employed in the present work (Supporting Information Pages S6–S8 and supplemental Fig. S10). However, due to the sometimes large differences in abundances for a given glycosylation sites, some GADS can show significant differences. The median GADS similarity score for a single glycosylation site among different proteases is 875, which is slightly lower than that found for repeat measurements of a given peptide from the same protease (>900). These differences in similarity scores are generally a consequence of different numbers of glycopeptide identifications for peptides generated from different proteases, which in turn is most likely a result of different abundances, with GADS having smaller numbers of identifications being more variable (Fig. 3). However, a similarity score above 800 is confirmatory and GADS will appear similar (Fig. 5). Some illustrations of larger differences are shown in Table 3 and supplemental Fig. S11 and can cast doubt on the accuracy of the distribution. The number of total glycopeptides identified for each protease combination varied, depending on the protein (supplemental Table S3).

**Fig. 6:**
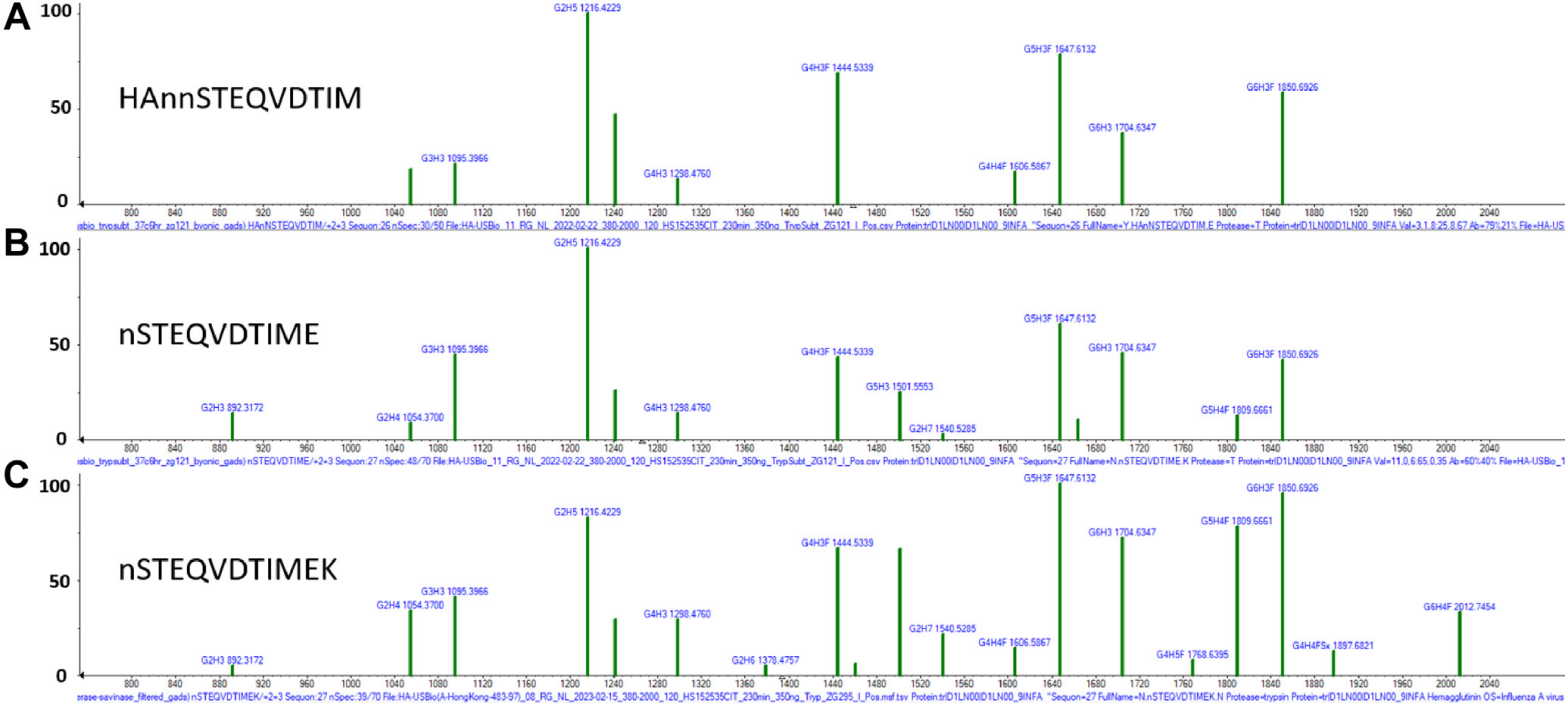
**Esperase and subtilisin proteases cleave adjacent asparagine.** Four proteases not widely used in proteomics and often classified as “nonspecific” were tested to determine if they might cleave between adjacent possible N-linked glycosylated asparagine sites in HA-HK97. GADS are shown for (*A*) a peptide from trypsin + subtilisin digestion containing both sequons. *B*, trypsin + subtilisin digestion in which the second glycosylation site was detected, but not the first. *C*, esperase digestion also in which only the second glycosylation site was detected. GADS compared are from protein HA-HK97 at glycosylation sites 26 and 27. The match of the three GADS is consistent with an unoccupied asparagine N terminal to the occupied site. MSfragger-glyco (31) in nonselective mode was employed for (*B*) and (*C*). GADS, glycopeptide abundance distribution spectra; HA, hemagglutinin; NA, neuraminidase.

Four nonspecific proteases were utilized in an effort to isolate the adjacent potential glycosylated sites in protein HA-HK97. Either or both of these neighboring sites (NNST) are potentially occupied and therefore site-specific GADS could not be determined unless they were separated by a cleavage between the adjacent asparagines. Both electron transfer dissociation and hybrid electron transfer dissociation with higher energy collisional dissociation were unsuccessful in isolating these glycosylation sites, with no c and z fragments involving breakage of the N-N bond. Therefore, the rarely used proteases subtilisin, neutrase, esperase, and savinase were employed in an effort to isolate the glycosylation sites. While these are generally not used in proteomics, apparently due to their unpredictable or nonselective cleavage, both subtilisin and esperase did in fact cleave these adjacent asparagine residues, generating abundant glycosylated peptides containing the second asparagine. However, no peptides containing only the first asparagine, glycosylated or not, were identified. Since peptides containing both potential glycosylated sites were found to contain the same glycan distribution as the peptides containing the isolated site (Fig. 6), and no unidentified glycopeptides were found in the chromatographic region where they eluted, only the second asparagine appears to be occupied. The removal of the apparently unmodified asparagine had little effect on the glycosylation distribution (similarity score = 872), which is similar to the degree of variation observed from the changing peptide landscape due to different proteases. If both sites had been occupied, the observed glycan mass would be the sum of masses of glycans on the two sites and would generally not correspond to glycans in the library. For further confirmation of occupancy, deglycosylation was performed using PNGase F and deamidation was searched for. This indicated that the second site is occupied (deamidated), but again no reliable identifications were found for peptides containing the first glycosylation site. The evidence points to the likelihood that the first glycosylation site is unoccupied, consistent with the probable steric hindrance that may have prevented double occupancy. While successful isolation was achieved for adjacent sites using subtilisin and esperase, this was only tested for the protein HA-HK97. Differing results for other proteins containing adjacent glycosylation sites may occur and are planned for study in future studies.

### Variation Between Suppliers

**Fig. 7:**
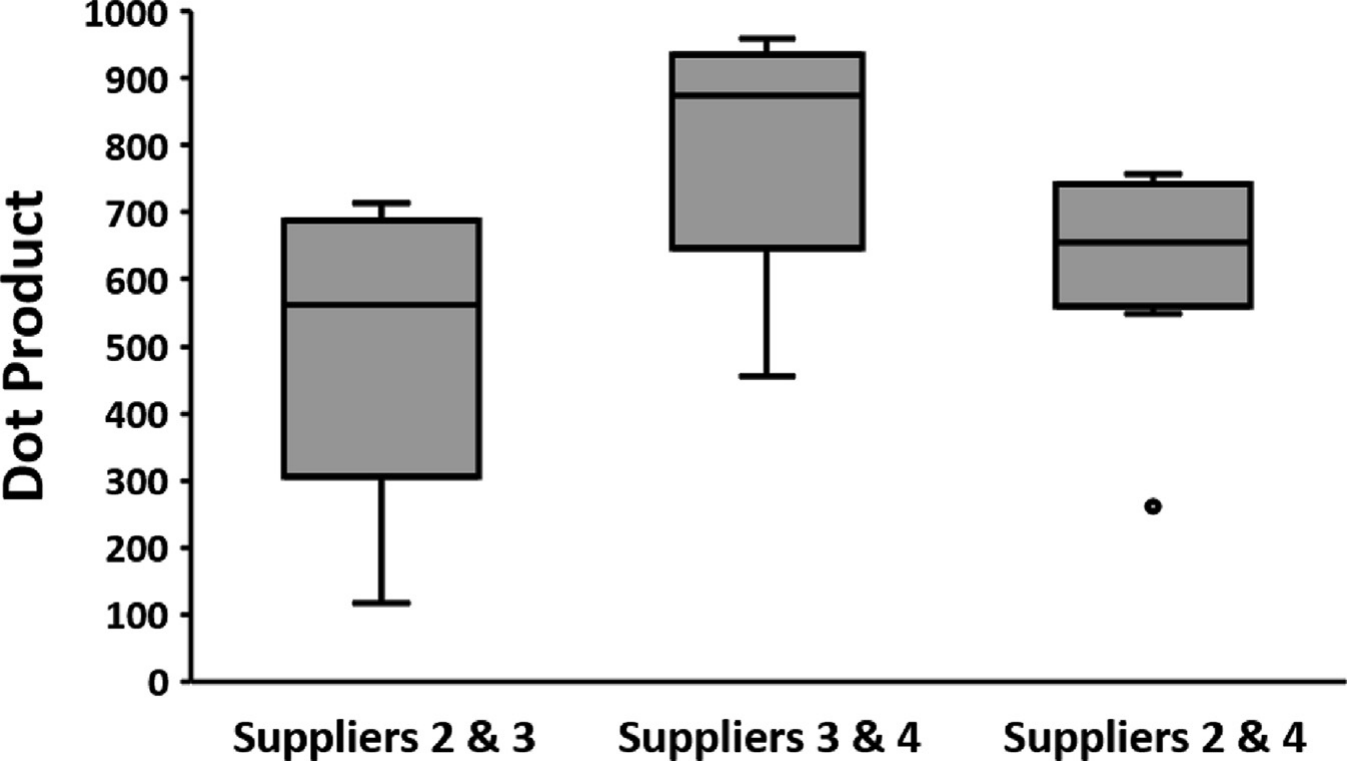
***Box and whisker plot* of intersupplier glycosylation comparisons.** Similarity scores were compared for three different suppliers for matching glycosylation sites in proteins HA-HK97 and NA-TH04. The *y*-axis represents similarity as calculated by the dot product. *Box and whisker plots* represent the four quartiles of the data distributions. GADS, glycopeptide abundance distribution spectra; HA, hemagglutinin; NA, neuraminidase.

While experiments have been conducted to elucidate the glycosylation differences from different sources of a protein (11, 21), only recently has work been reported to distinguish between different suppliers creating the same proteins from the same cell line (25). In this section, we build on this previous work to compare the same pair of influenza proteins (HA-HK97 and NA-TH04) from three suppliers, all of which used the HEK293 cell line. The median similarity score for variation between suppliers is 781 with an interquartile range of 679 to 847 (Fig. 3 and supplemental Table S4), which is significantly lower than the interquartile range of 857 to 916 for proteins from the same supplier (Fig. 3). Variation in glycosylation between suppliers is the largest drop in similarity among the factors considered so far and is consistent with earlier findings for the SARS-CoV-2 spike protein (25). Clearly, differing lab practices for the creation of recombinant proteins can create significantly different glycosylation pattens despite their formal equivalence in expression methods. Suppliers 3 and 4 are the most similar in glycosylation, with a median similarity score of 874 between equivalent peptides. However, supplier 2 had a more distinct glycan distribution compared to the other two, with the higher mass multisialylated glycans being in higher abundance (Figs. 2 and 7).

### Variation Between Different Glycosylation Sites Within the Same Protein (Meta-Heterogeneity)

Distributions of N-glycans can vary radically between different sites within the same protein (32). The diversity of these distributions has been termed “meta-heterogeneity” (9). It has been observed that while each glycosylation site of a protein may have a unique glycan distribution, there can be substantial similarities between sites in a given protein (9, 33). Comparisons of GADS from a single protein enable the direct quantitative assessment of this similarity of any two sites. Unsurprisingly, results here indicate that glycosylation distributions are more dissimilar between different sites within the same protein than the factors considered so far. They also have the largest range of similarity scores. The median similarity score is 643 with an interquartile range between 535 and 731. This range is broad and there are many values that are above a score of 700 and below a score of 500. As with previous conditions, glycans that are most shared between glycosylation sites include high mannose and core-fucosylated biantennary and triantennary complex glycans (Table 3). Glycans that are only observed at one glycosylation site include G6H3F at position 126 in protein NA-TH04, G5H4FS2 at position 170 in protein HA-HK97, and G3H5FS at position 104 in protein HA-CA09 (Table 3). The most similar intrastrain glycosylation sites in terms of glycan distributions are positions 146 and 455 in protein NA-AZ08 (similarity score = 962) and positions 302 and 500 in protein HA-HK97 (922). The most dissimilar intrastrain glycosylation sites are positions 71 and 104 in protein HA-NC99 (133) (supplemental Figs. S12–S14).

### Variation Between Influenza Strains

**Fig. 8:**
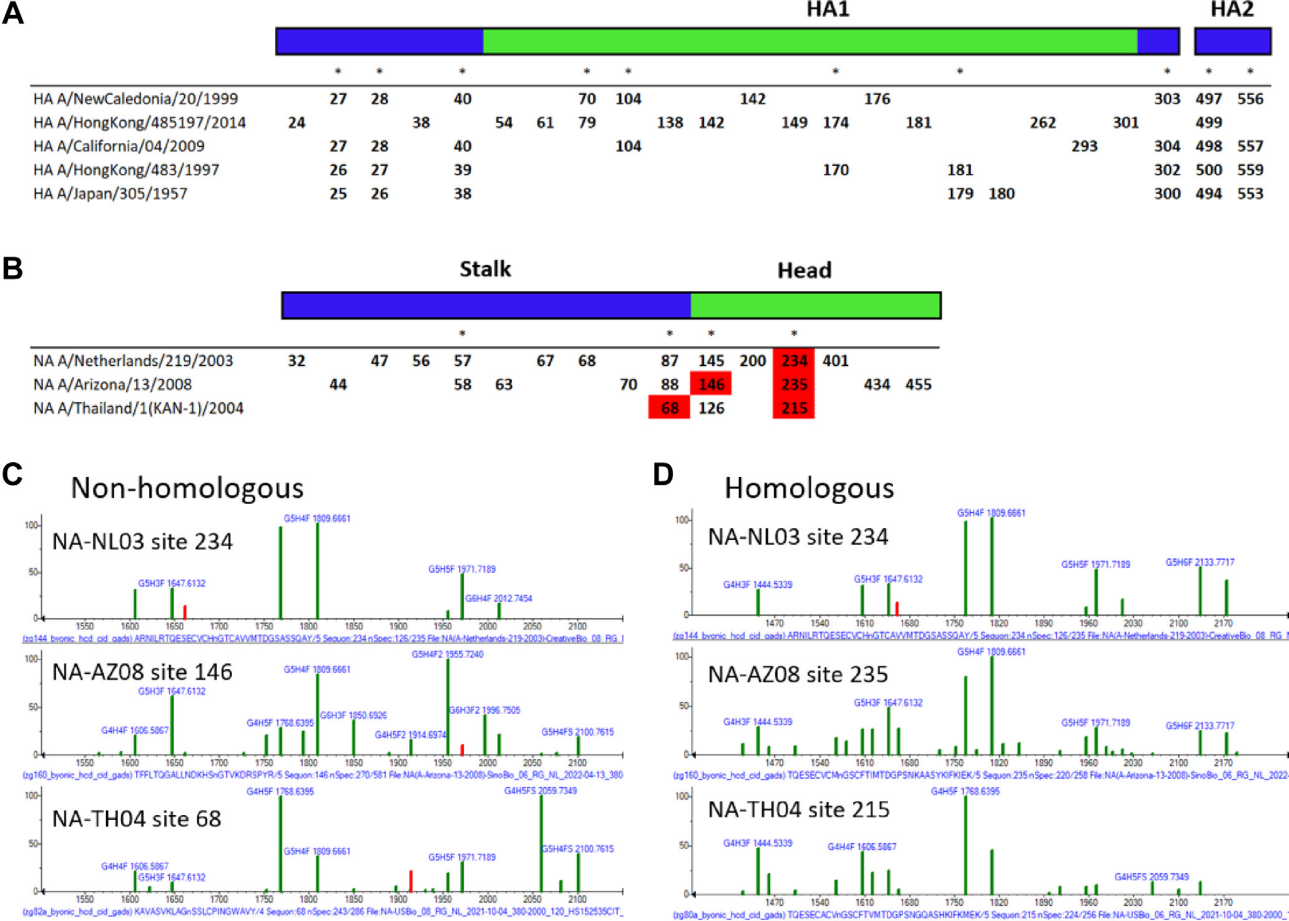
**Homologous and non-homologous glycosylation sites for HA and NA proteins.** Glycosylation sites for different strains of the same protein can be conserved (homologous sequences) or divergent (nonhomologous sequences). *A*, sequences for five HA strains. *Asterisks* indicate alignment locations of homologous sites. The *green region* represents the head of HA, and the *blue region* represents the stalk. *B*, sequence construct for three NA strains where sites highlighted in *red* denote comparisons made in subfigures (*C* and *D*). *C*, three GADS from nearby but nonhomologous sites on three different strains. *D*, three GADS from a homologous region on three different strains. GADS, glycopeptide abundance distribution spectra; HA, hemagglutinin; NA, neuraminidase.

Glycan variation between strains is of special interest since it relates to glycosylation changes with viral mutation and has been of concern in other studies (11, 12, 34). Since influenza is a rapidly mutating virus, this type of variation is of particular concern. We make comparisons of between glycosylation sites that overlap after sequence alignment of two proteins as well as nonaligned sites after protein sequence alignment (Fig. 8, and supplemental Figs. S1 and S2). Similar glycosylation for corresponding sites on different variants indicates conservation of glycosylation despite evolutionary changes in sequences. Despite the great deal of effort spent in tracing these evolutionary changes and the creation and loss of glycosylation sites (35), this appears to most detailed examination of changes in their glycome (36).

**Fig. 9:**
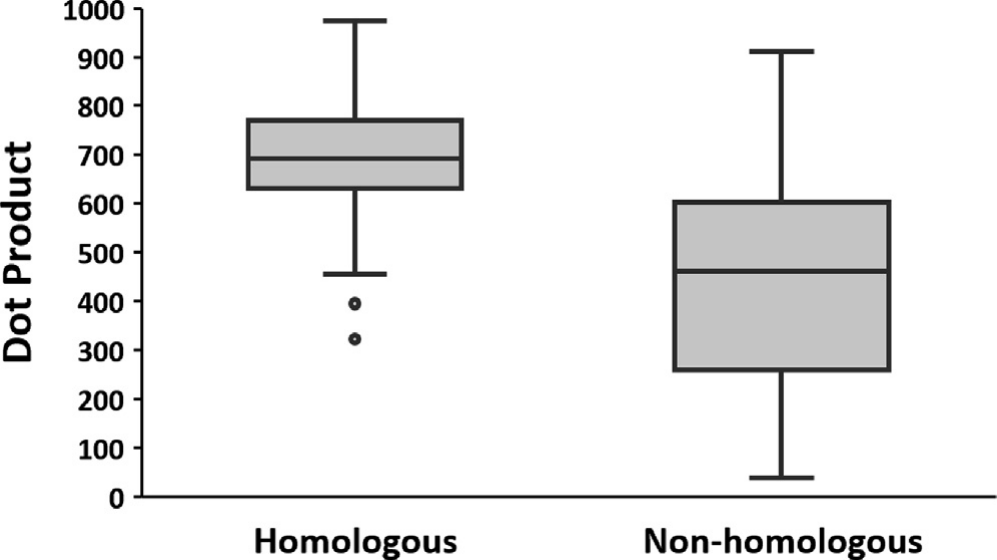
**Similarity in glycosylation of interstrain sites for homologous and nonhomologous regions.** GADS similarity was compared between homologous and nonhomologous glycosylation sites in different strains of HA and NA as depicted in Figure 8. Homologous regions are represented with an *asterisk* in Figure 8. The *y*-axis represents similarity as calculated by the dot product. *Box and whisker plots* represent the four quartiles of the data distribution. GADS, glycopeptide abundance distribution spectra; HA, hemagglutinin; NA, neuraminidase.

Overall, the median similarity score for interstrain GADS comparisons is 383 with an interquartile range of 286 to 555. These are very low scores compared the corresponding range of 535 to 731 for intrastrain comparisons. Note that the overlap of interquartile scores for these two groups is only 2%. Intrastrain similarity yields the largest range of scores among all factors tested thus far. However, for the comparison of homologous glycosylation sites between different strains, the median similarity score is 692 with an interquartile range of 631 to 770 while for nearby nonhomologous sites, the median similarity score is very low at 461 with an interquartile range of 260 to 602 (Fig. 9). This involved 332 comparisons between homologous sites and 312 comparisons between nonhomologous sites. These results indicate that glycosylation distributions of sites with sequence homology are far more similar than are nonhomologous sites.

In terms of glycan-level differences between strains, there are some that are highly conserved and some that are more strain-specific. The most abundant glycans (>20% relative abundance in GADS) and consistently observed between HA and NA strains include core-fucosylated biantennary and triantennary complex glycans (Table 3). Also, certain glycans are highly abundant in only one strain. For example, G2H9 is a major component for HA-HK14, G3H3 for HA-HK97, while G6H7FS2 is characteristic for HA-JP57. The presence of such unique glycans highlights the unpredictability of strain-to-strain glycosylation.

## Discussion

The measurement of site-specific glycosylation by mass spectrometry is now widely used to characterize glycan distributions (37, 38, 39, 40). However, these measurements and their subsequent analysis are known to be challenging due to oftentimes low signal levels and false positive risk (25, 40, 41). Relatively little has been reported concerning the actual variability encountered in these measurements. Not unexpectedly, distributions of glycopeptides with fewer identifications are more variable, less complete, and more prone to misidentifications. This is the principal source of experimental variability and uncertainty as expressed in supplemental Fig. S4. In view of the importance of these measurements (7, 42, 43), we have focused not only on reliable determinations of these distributions but also on the measurement of their variation. Key to this analysis is a newly developed data analysis method which records results and their uncertainties—GADS. This is employed to objectively measure the variation in mass spectrometry-based methods of site-specific glycosylation profiling caused by experimental, instrument, and protein synthesis factors. These measurements were performed on proteins of significant interest in glycobiology—influenza glycoproteins hemagglutinin (HA) and neuraminidase (NA). Influenza rapidly mutates and therefore has a large variety of strains and, consequently, a variety of glycosylation sites, some conserved and some novel. The expression source was the widely used HEK293 cell line due to its established methods of recombinant expression and, being human in origin, the expectation that could simulate glycan distributions in humans. However, the present data clearly show that there can be great variability even using a single cell line when the same protein sequence is synthesized in different labs.

The factors that had a relatively low degree of variation (median similarity scores 875–957) include injection replicates, digestion replicates, supplier batches, and the use of different proteases. These factors were used to confirm the accuracy of distributions and allow the quantitative assessment of reproducibility. While these factors could lead to noticeable variations in site-specific measurements, similarity scores were mostly above 800, indicating overall confidence in the measurement. Note that a similarity score above 800 is generally considered to be good agreement between mass spectra in widely applied mass spectral library searching methods (44). The multiple sources of variation are qualitatively understood and the oftentimes low abundance of glycopeptides and the limited number of monosaccharide masses can make confident identification difficult. The use of multiple proteases serves to (1) increase the likelihood that abundant glycopeptides for each glycosylated site is generated; (2) increase the likelihood that each glycosylated site is present in a single peptide sequence; (3) confirm reliability for a glycosylation site when multiple GADS are generated for different peptides containing that site; and (4) increase confidence when the same peptide in generated by different protease combinations (45). These factors add considerable confidence to reported glycosylation profiles and to each reported glycan, and limit false positive interference (46), as well as allow scrutiny of the relative abundance of each reported glycan.

Since this work examines multiple sources of variability, some discussion of their origin is warranted. For injection replicates, some potential sources are stochastic sampling, nanoflow retention variation, instrument contamination and carryover, sample stability, and inadvertent variations in injection amounts (47, 48, 49). Though the specific sources of variation for injection replicates and the extent of their contribution cannot be identified, it is likely that stochastic sampling of the data-dependent acquisition method is a key contributor due to all other factors being held constant between injections. Variation between digestion replicates adds factors related to sample digestion and processing, including denaturation and peptide purification steps. Finally, the multiple types of protease digestion and their sensitivity to chemical conditions is another inherent source of variability. These differences in digestion efficiency and ionization efficiency often lead to different numbers of glycopeptide abundance and identifications, which is a principal factor affecting GADS reproducibility (supplemental Fig. S4).

The factors with the highest degrees of actual variation in glycosylation (median similarity scores 195–781) include different suppliers, intrastrain, and interstrain heterogeneity. The precise origin of these specific variations is generally obscure due to the multiple factors involved in protein synthesis and the fact that glycosylation dependence on protein structure is largely unpredictable (50). The large degree of variation in glycosylation between different sites of the same protein can be attributed mainly to factors to which glycosylation depends on, which is well documented (9). An unexplored possible reason for vendor differences may be due to the different “trimming” done at the protein termini. While there are many biological factors that could be responsible for the differences in glycosylation between sites, other experimental and analytical influences could contribute to the detection of glycosylation between different sites on the same protein.

GADS similarity scores were shown to be very discriminating when comparing distributions due to either measurement variation or experimental and biological variation. These similarity scores ranged from a median similarity score of 957 for replicate injections to a score of 383 for interstrain differences. Variations were even greater comparing GADS for unrelated proteins (4), where HA was compared with seven unrelated glycoproteins (supplemental Fig. S15). For these comparisons, similarity values ranged between near zero and 971 for certain high mannose sites, with a median value of 195 with an interquartile range of 100 to 361 (Fig. 3). This low level of similarity could be due to a lack of relatedness but could also be due to the expression source as HA was expressed in recombinant HEK293, while the other proteins were expressed native in human breast milk and plasma. Glycosylation comparisons between HA and another viral recombinant glycoprotein, SARS-CoV-2 spike, shows more similarity (supplemental Fig. S16). HA and SARS-CoV-2 spike have somewhat similar overall distribution in glycans, with complex glycans being the most abundant type, and galactose-containing, sialyl-containing, and fucose-containing glycans also being abundant and differences between protein sources showing similar degrees of variation.

## Conclusion

This work presents detailed site-specific distributions for a number of influenza glycoproteins as well as an analysis of their variation due to both biological and experimental factors. These comparisons extensively apply an intuitive and quantitative method of reporting these distributions in terms of the recently developed library-compatible GADS representation (21). Using this method, we have examined in detail potentially significant sources of variation. GADS measured the high degree of reproducibility that can be obtained in site-specific measurements between reinjection, replicate digestion, and the different proteases employed when the underlying glycopeptides are abundant. The strong relationship with reproducible measurements and glycopeptide abundance, or spectral counts, was quantitatively demonstrated. These results demonstrate the broad utility of this method for future comparisons of glycosylation arising from biological factors such as protein source or sequence mutations. Results here also demonstrate, not unexpectedly, that GADS between different strains from the same influenza protein are highly variable, but not as variable as unrelated proteins. However, glycosylation sites on different strains of influenza sharing a conserved sequence are much more similar than nonconserved regions. While reproducibility in experimental and analytical conditions can be high, glycosylation can differ greatly between suppliers of the same protein as found in previous work in the SARS-CoV-2 proteins (25). Additionally, an examination of correlations between the GADS reported here indicated that the majority could be separated into four distinct classes. Also, a number of formally “nonspecific” proteases were examined in an effort to separate the two adjacent potential N-glycosylation sites that are commonly found in HA. This led to the finding that only the second site (NnST) was occupied but also indicated the two of these proteases, subtilisin and esperase, hold promise for uniquely selective proteolysis for hard-to-isolate glycosylation sites. In summary, these observations of reproducibility and variability provide a convenient and confident means of reporting site-specific glycan distributions as well as a practical method for future comparisons of these distributions for this highly variable, difficult to measure and largely unpredictable structural characteristic of glycoproteins.

## Data Availability

All FASTA and RAW data files containing glycopeptide HCD and ion-trap collision-induced dissociation spectra cited in this work, including from the Supporting Information section, are publicly available on ProteomeXchange (PXD042062) *via* the PRIDE partner repository (51, 52). The folder includes output data files from the Byonic search, such as search xlsx and csv files, which contain information on each MS^2^ spectrum as well as protein information. A complete list of data files is presented in supplemental Table S1. GADS and MS^2^ libraries are freely available for download at Chemdata.nist.gov (https://chemdata.nist.gov/dokuwiki/doku.php?id=peptidew:influenza_a_glycoproteins) which can be visualized using NIST MS Search, which is also available for download (https://chemdata.nist.gov/dokuwiki/doku.php?id=chemdata:downloads:start#nist_ms_search_program). This is a modified version of a widely distributed for use with mass spectral reference collections from NIST and other sources.

## Supplemental Data

This article contains supplemental data (53).
