# Supplementary material for: Variation of Site-Specific Glycosylation Profiles of Recombinant Influenza Glycoproteins
Source: Mol Cell Proteomics. 2024 Aug 10;23(9):100827. doi: 10.1016/j.mcpro.2024.100827 (PMC11417209; doi:10.1016/j.mcpro.2024.100827)
Supplement: Supplemental Document S1 [file mmc1.pdf]

Representative GADS for each protein (excluding adjacent sites)

<u>Hemagglutinin from strain A/Hong Kong/483/1997</u>

Glycosylation site 39

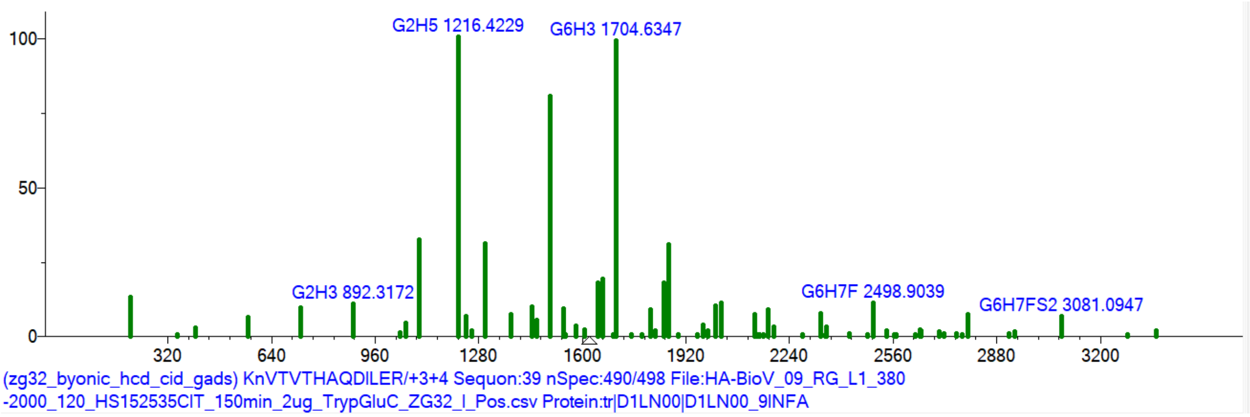

Glycosylation site 170

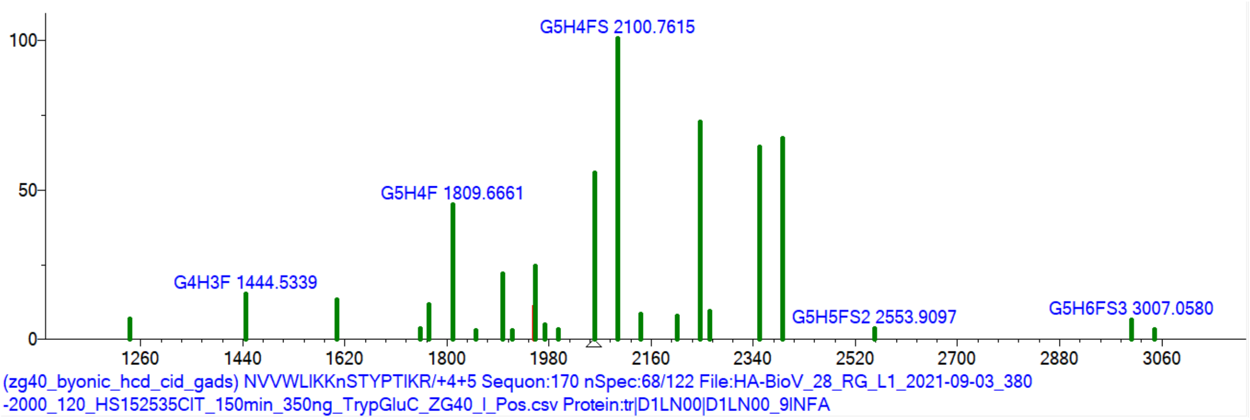

Glycosylation site 181

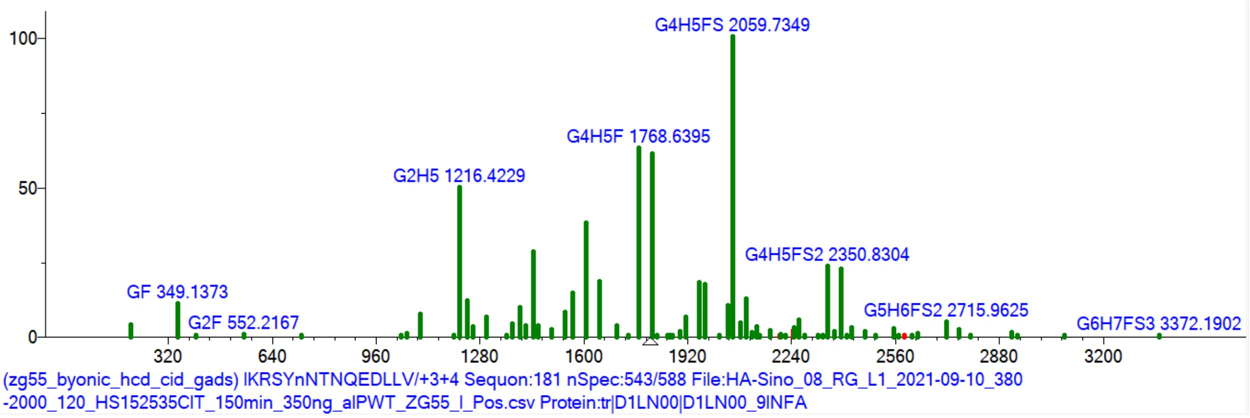

Glycosylation site 302

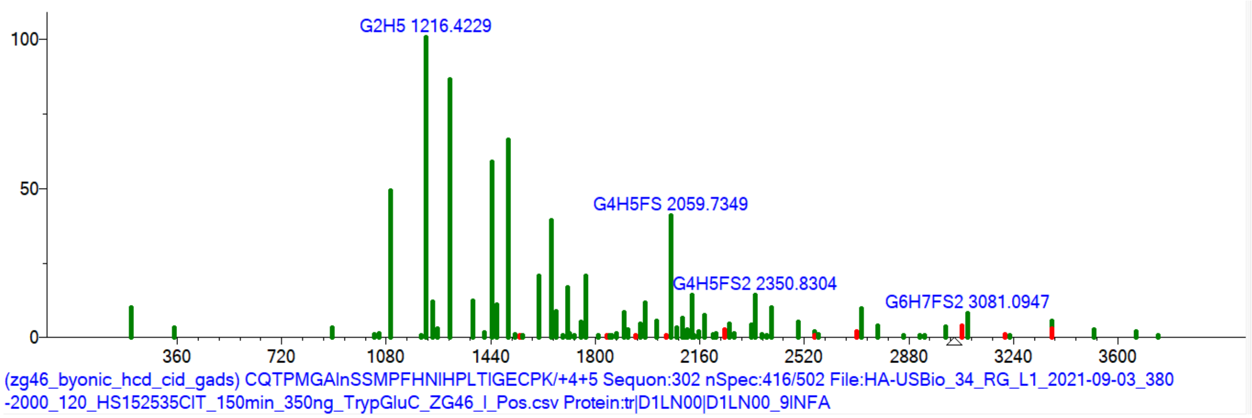

Glycosylation site 500

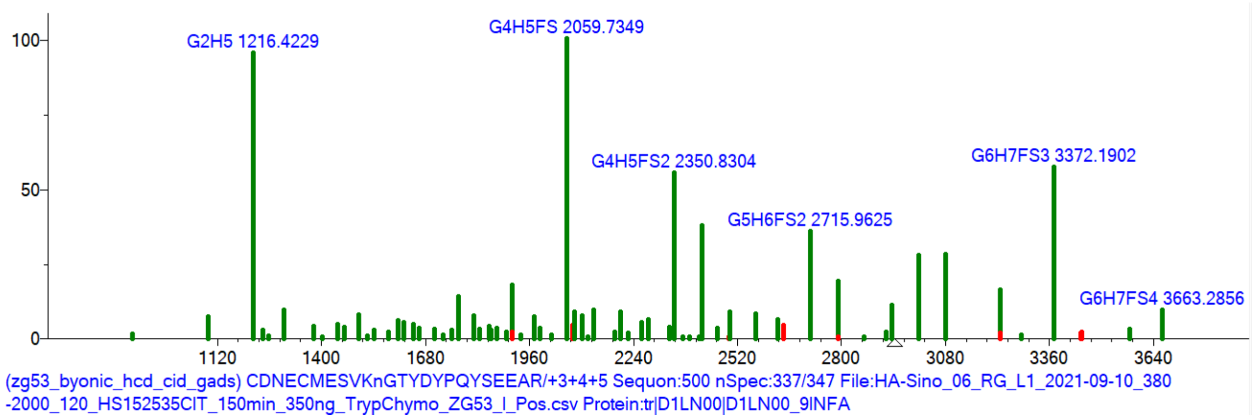

<u>Hemagglutinin from strain A/California/04/2009</u>

Glycosylation site 40

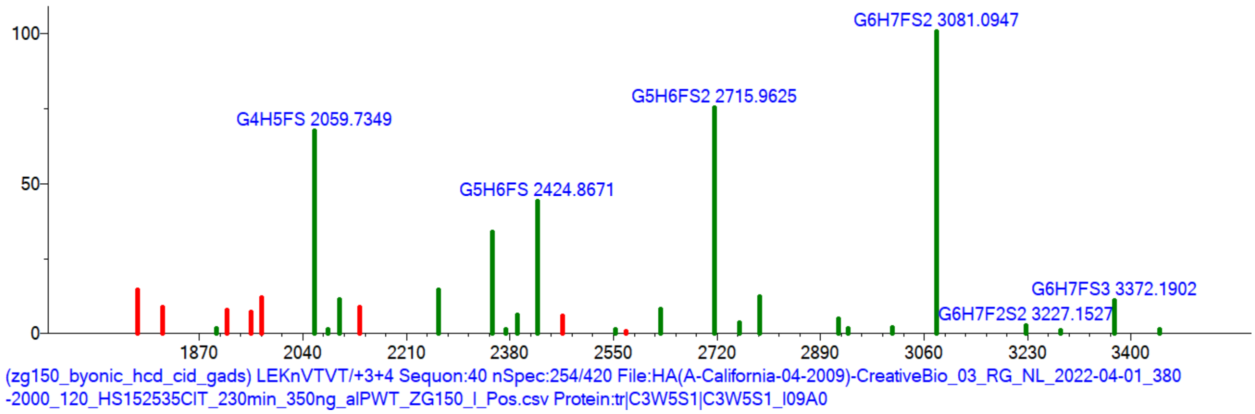

Glycosylation site 104

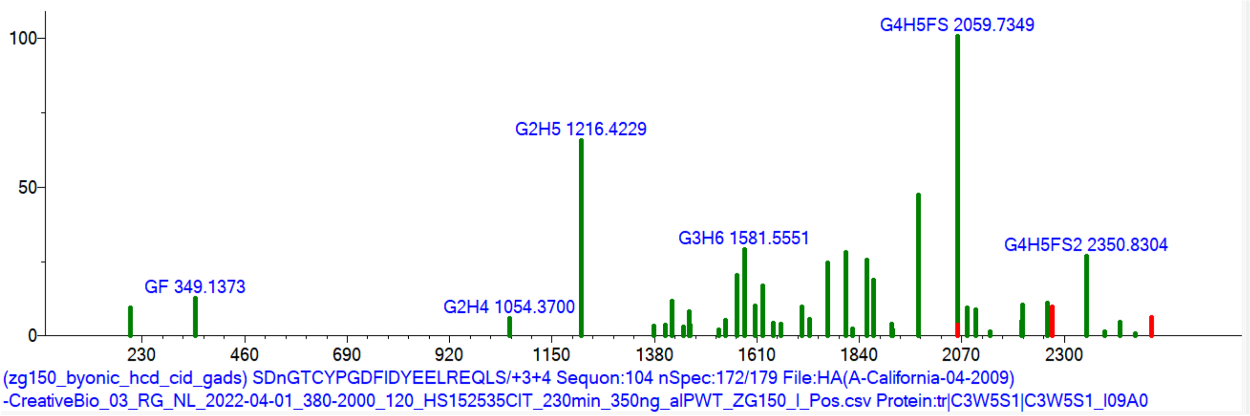

Glycosylation site 293

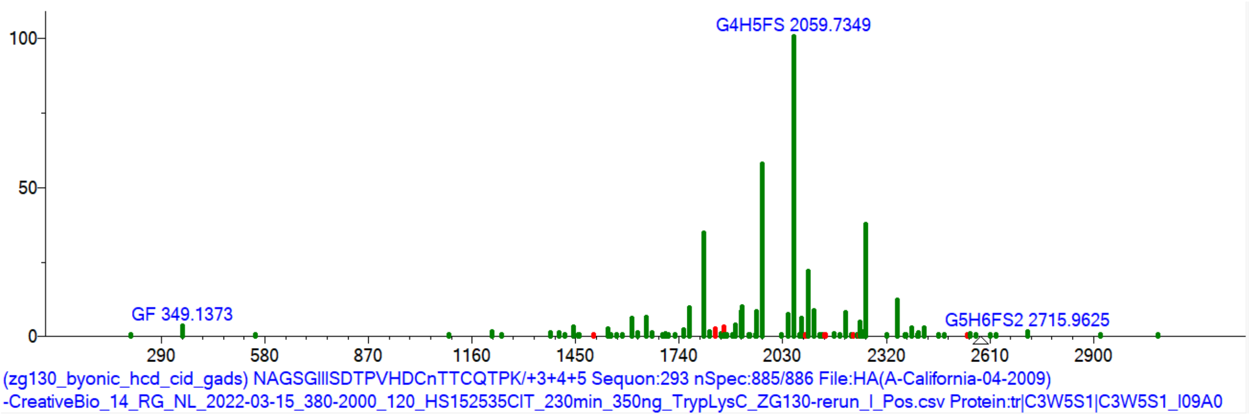

Glycosylation 304

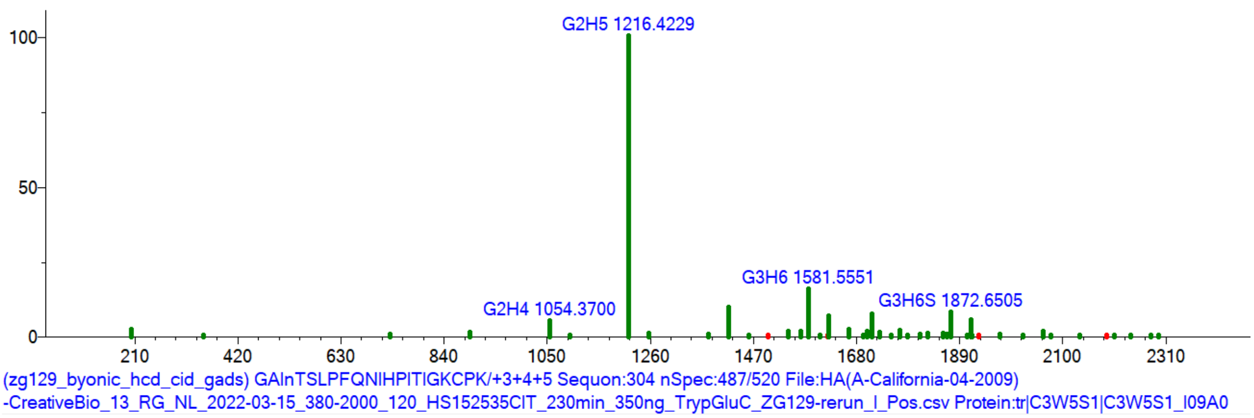

Glycosylation site 498

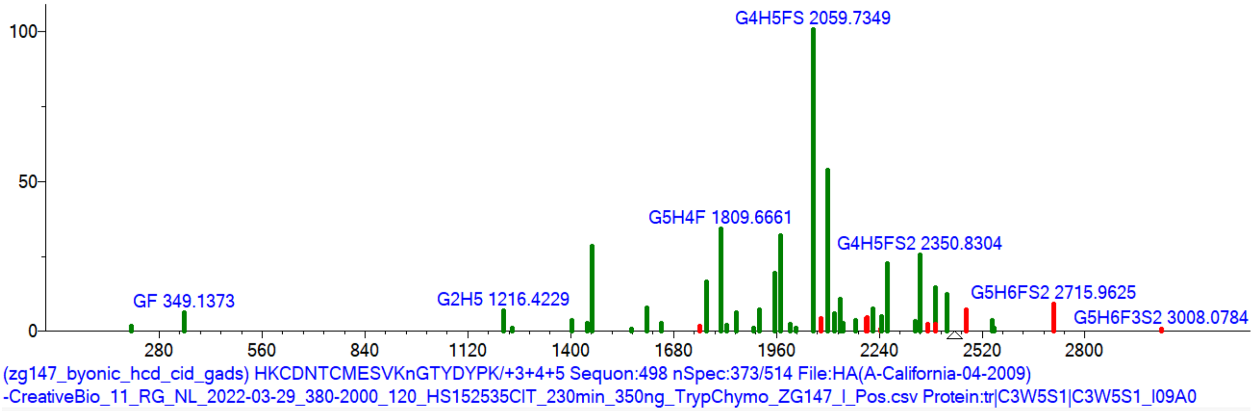

Hemagglutinin from strain A/Japan/305/1957

Glycosylation site 300

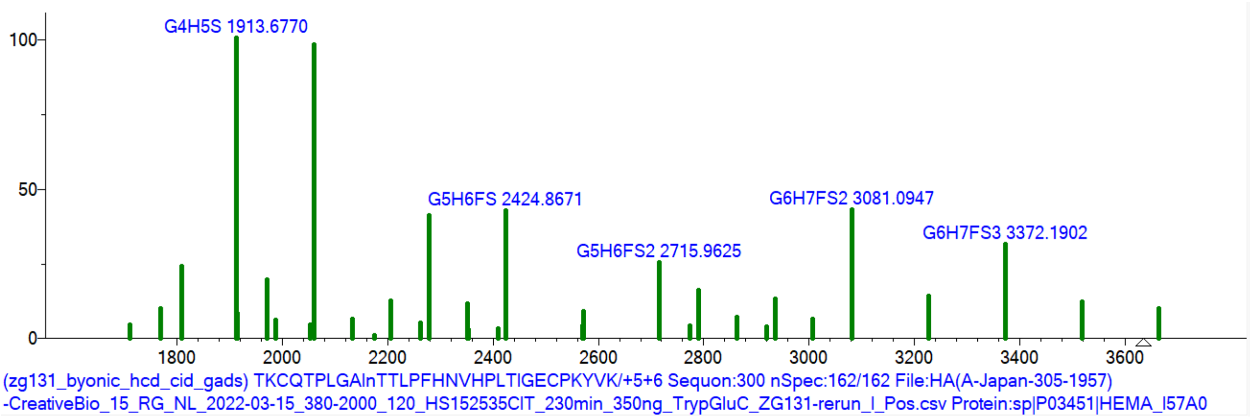

Glycosylation site 494

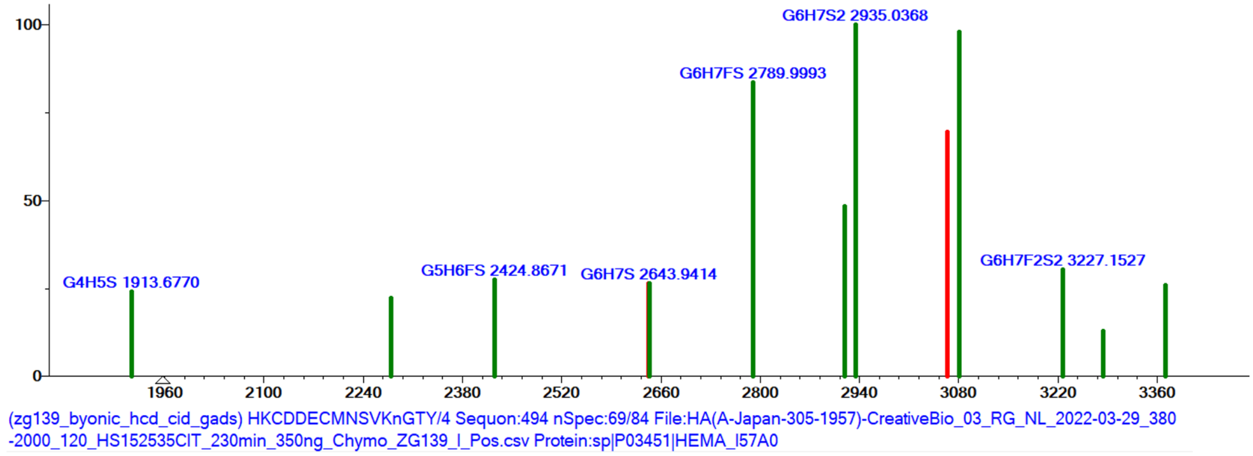

Hemagglutinin from strain A/New Caledonia/20/1999

Glycosylation site 40

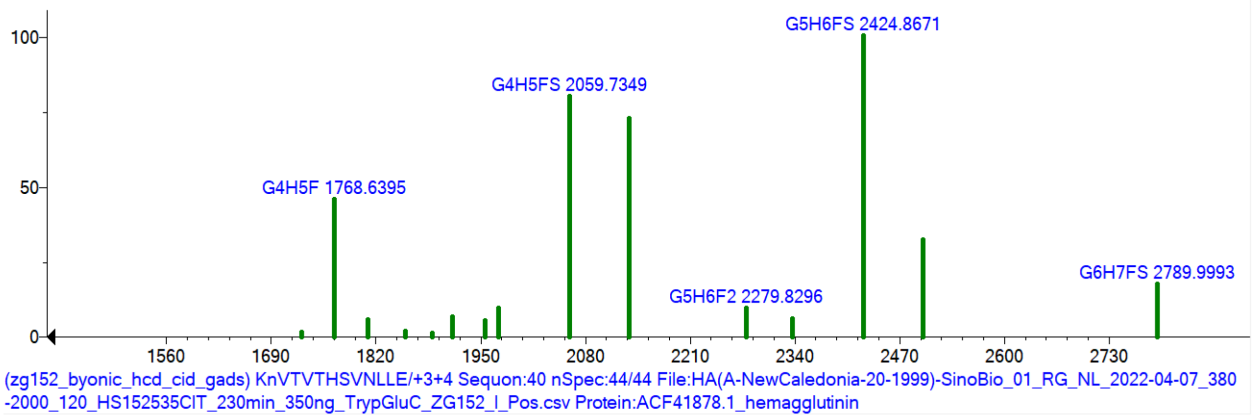

Glycosylation site 71

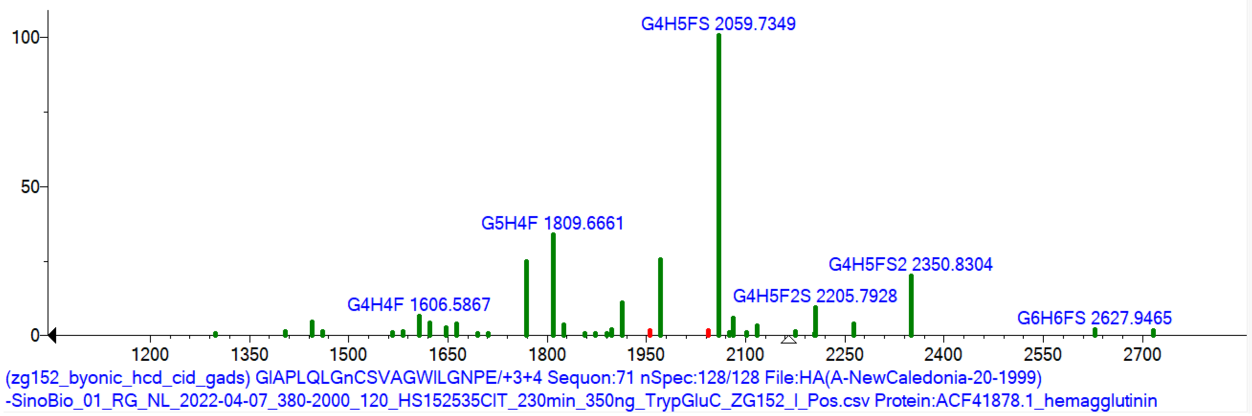

Glycosylation site 104

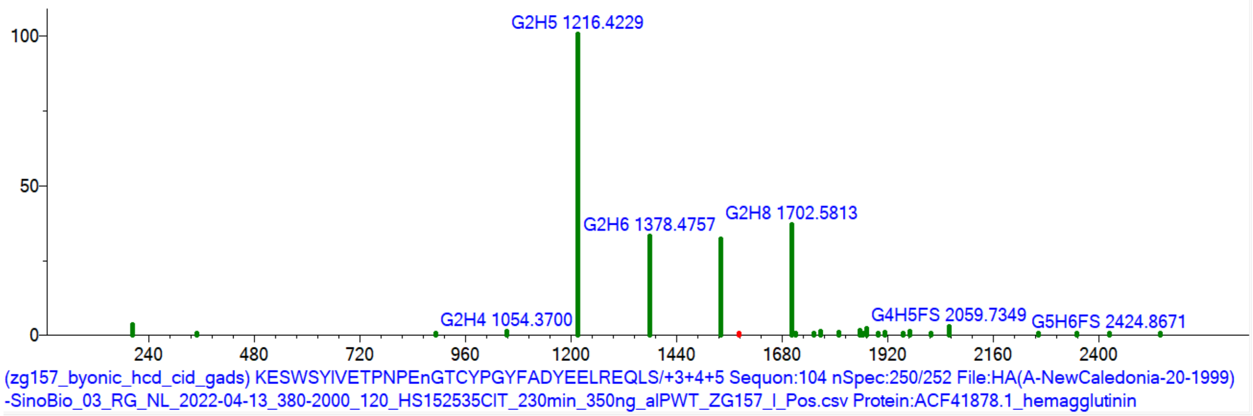

Glycosylation site 142

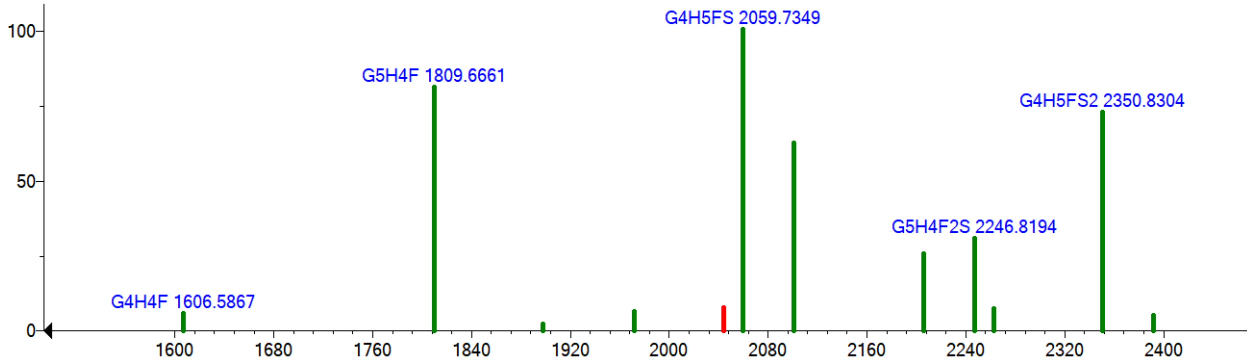

(zg152_byonic_hcd_cid_gads) IFPKESSWPnHTVTGVSASCSHNGK/+4+5 Sequon:142 nSpec:60/60 File:HA(A-NewCaledonia-20-1999)
-SinoBio_01_RG_NL_2022-04-07_380-2000_120_HS152535CIT_230min_350ng_TrypGluC_ZG152_I_Pos.csv Protein:ACF41878.1_hemagglutinin

Glycosylation site 176

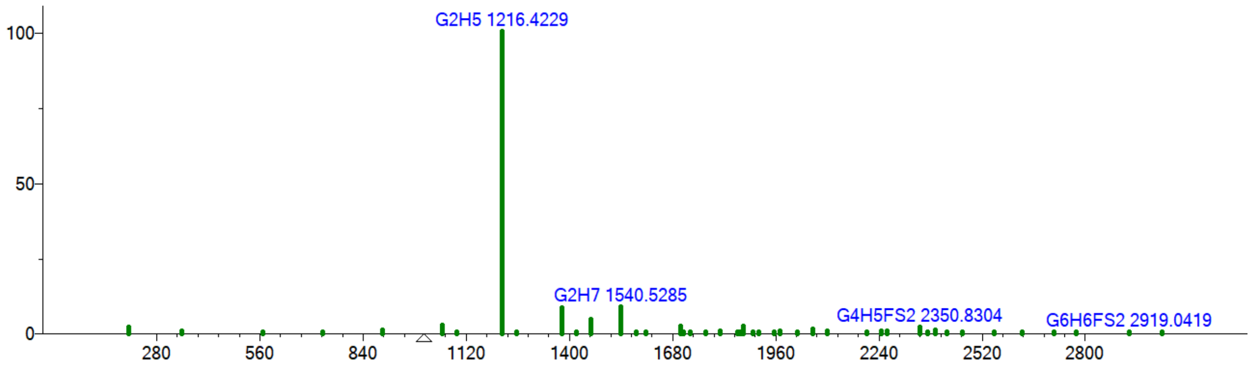

(zg154_byonic_hcd_cid_gads) NGLYPnLSK/+2+3 Sequon:176 nSpec:237/237 File:HA(A-NewCaledonia-20-1999)-SinoBio_03_RG_NL_2022-04-07_380
-2000_120_HS152535CIT_230min_350ng_TrypChymo_ZG154_I_Pos.csv Protein:ACF41878.1_hemagglutinin

Glycosylation site 303

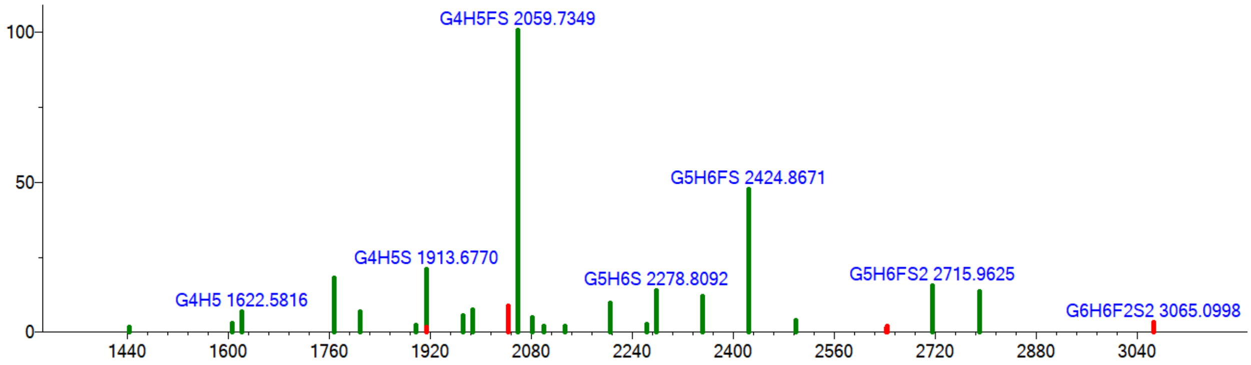

(zg152_byonic_hcd_cid_gads) CDAKCQTPQGAInSSLPFQNVHPVTIGECPK/+4+5 Sequon:303 nSpec:128/128 File:HA(A-NewCaledonia-20-1999)
-SinoBio_01_RG_NL_2022-04-07_380-2000_120_HS152535CIT_230min_350ng_TrypGluC_ZG152_I_Pos.csv Protein:ACF41878.1_hemagglutinin

Glycosylation site 497

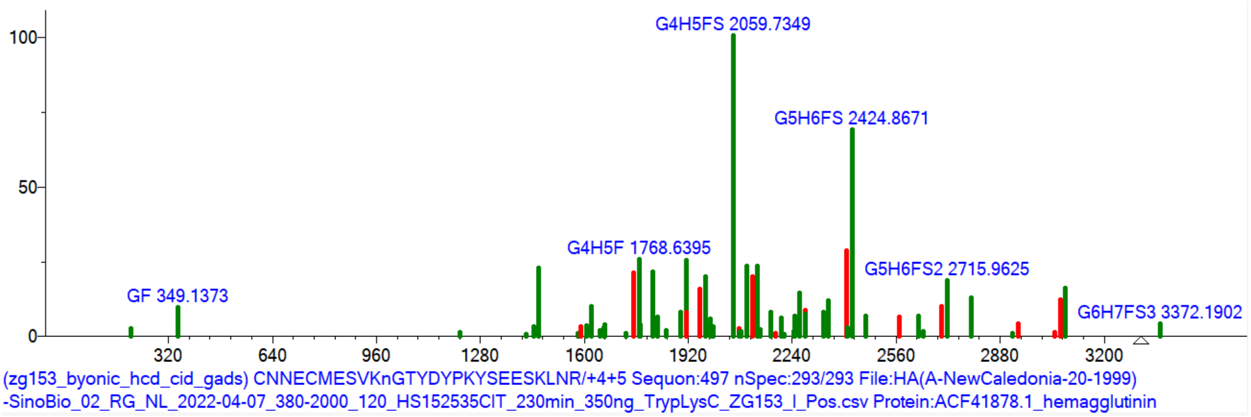

Hemagglutinin from strain A/Hong Kong/485197/2014

Glycosylation site 38

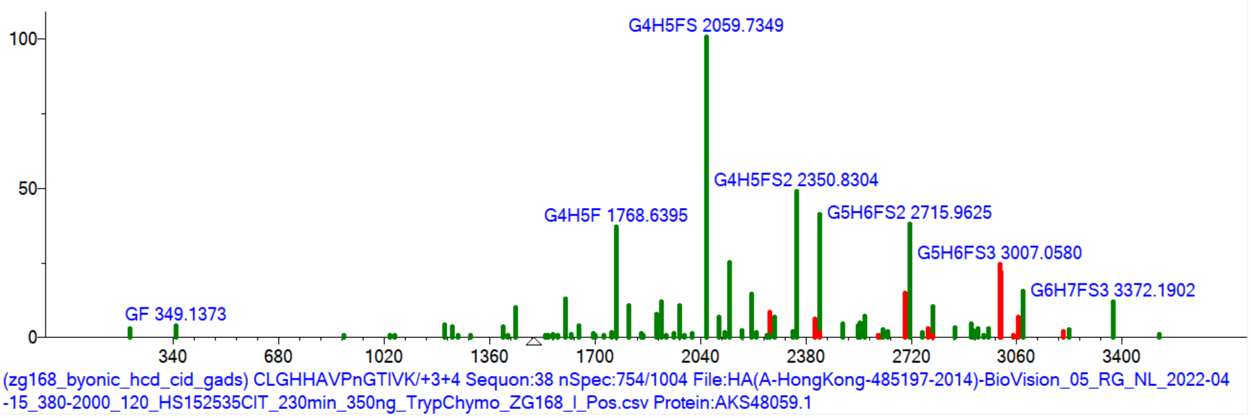

Glycosylation site 54

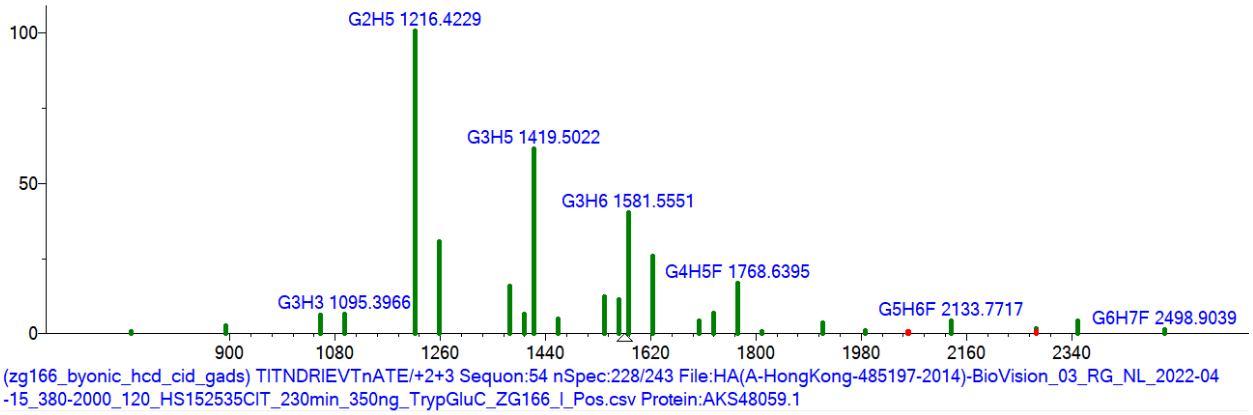

Glycosylation site 61

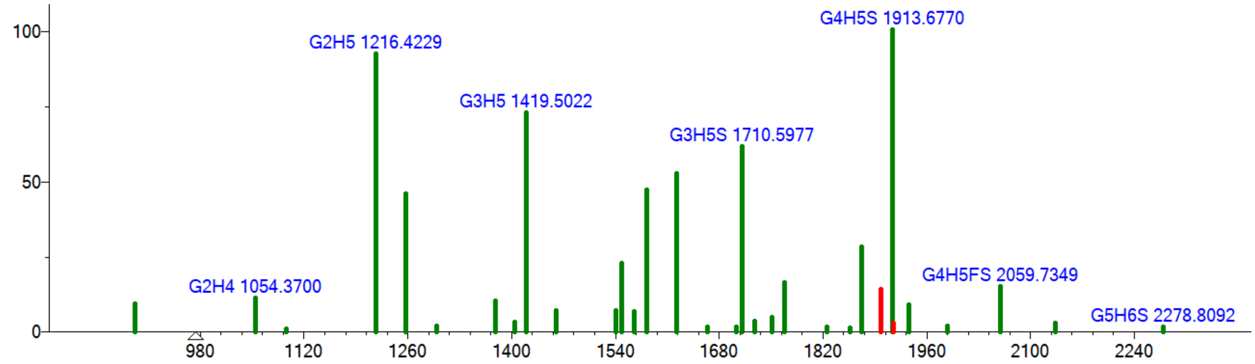

(zg171_byonic_hcd_cid_gads) NATELVQnS/+2+3 Sequon:61 nSpec:282/308 File:HA(A-HongKong-485197-2014)-BioVision_08_RG_NL_2022-04-15_380-2000_120_HS152535CIT_230min_350ng_alPWT_ZG171_I_Pos.csv Protein:AKS48059.1

Glycosylation site 262

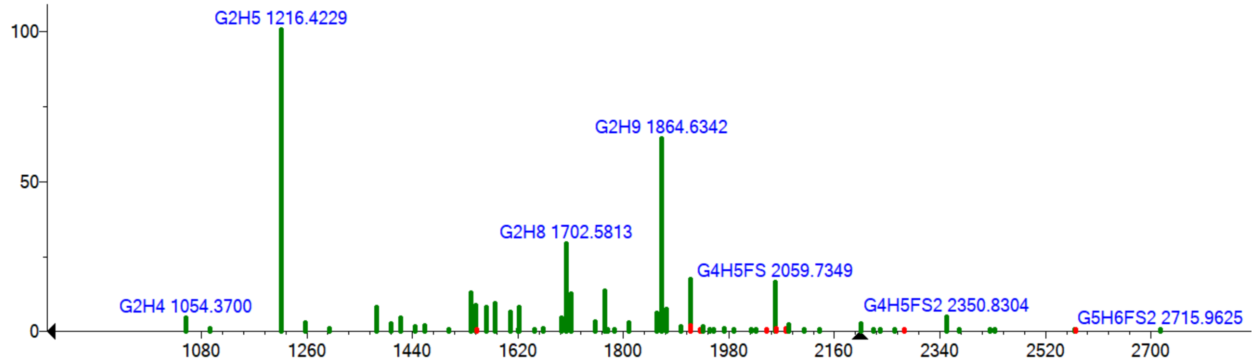

(zg168_byonic_hcd_cid_gads) TIVKPGDILLInSTGNLIAPR/+3+4+5 Sequon:262 nSpec:895/979 File:HA(A-HongKong-485197-2014)-BioVision_05_RG_NL_2022-04-15_380-2000_120_HS152535CIT_230min_350ng_TrypChymo_ZG168_I_Pos.csv Protein:AKS48059.1

Glycosylation site 301

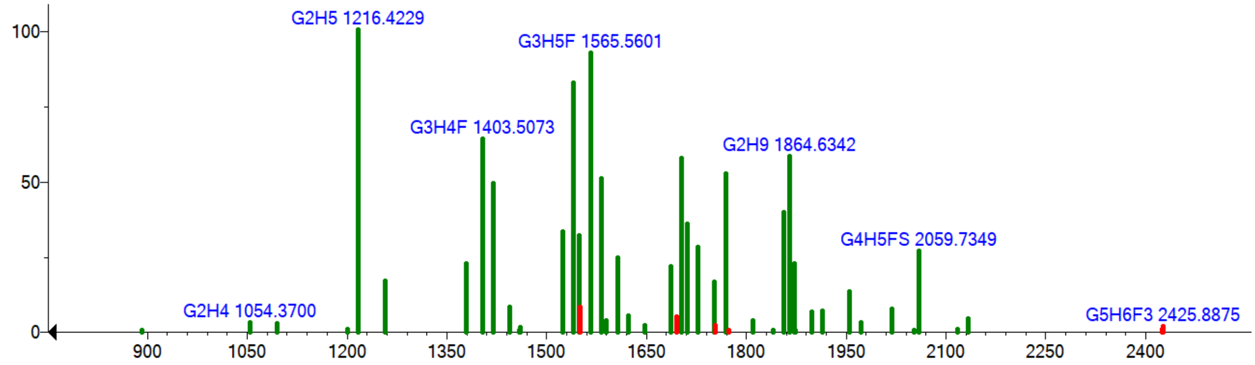

(zg168_byonic_hcd_cid_gads) CKSECITPnGSIPNDKPFQNVNR/+3+4+5 Sequon:301 nSpec:570/648 File:HA(A-HongKong-485197-2014)-BioVision_05_RG_NL_2022-04-15_380-2000_120_HS152535CIT_230min_350ng_TrypChymo_ZG168_I_Pos.csv Protein:AKS48059.1

Glycosylation site 499

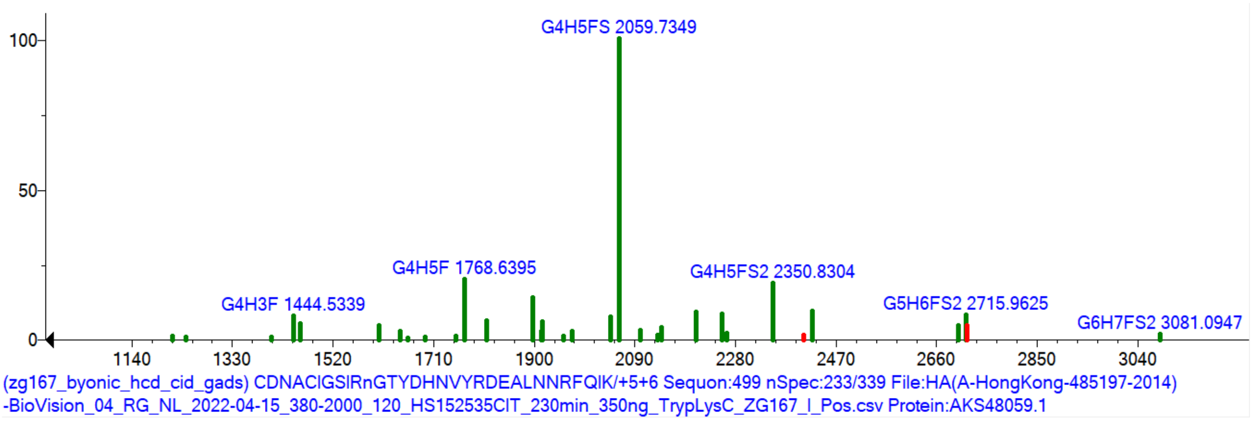

Neuraminidase from strain A/Thailand/1(KAN-1)/2004

Glycosylation site 68

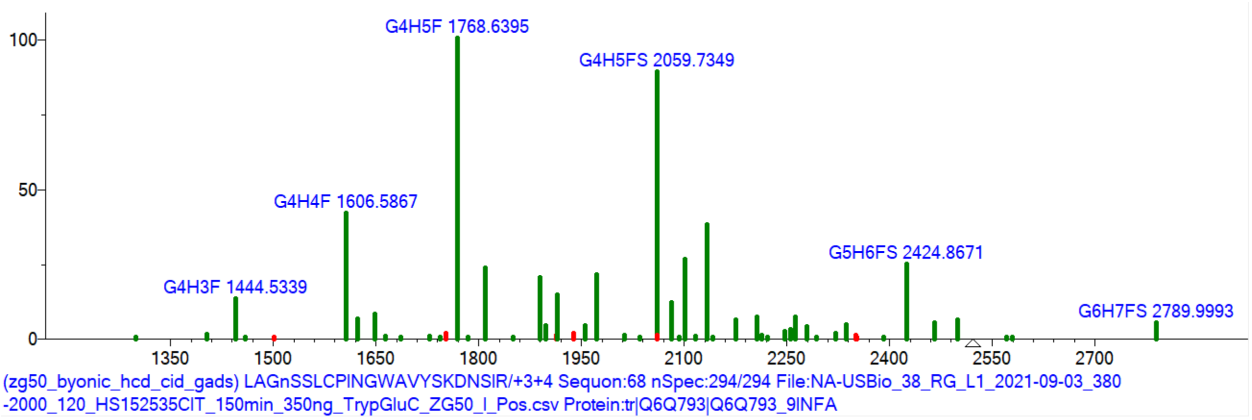

Glycosylation site 126

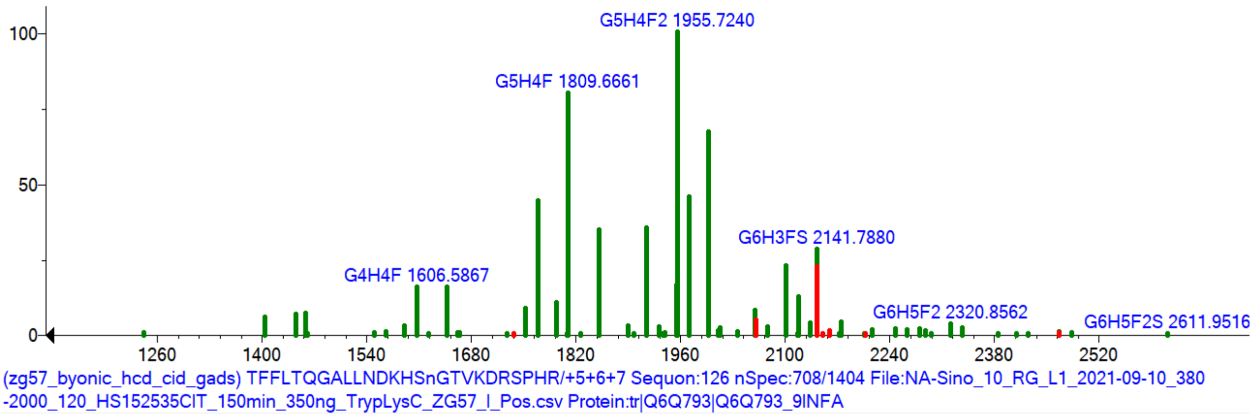

Glycosylation site 215

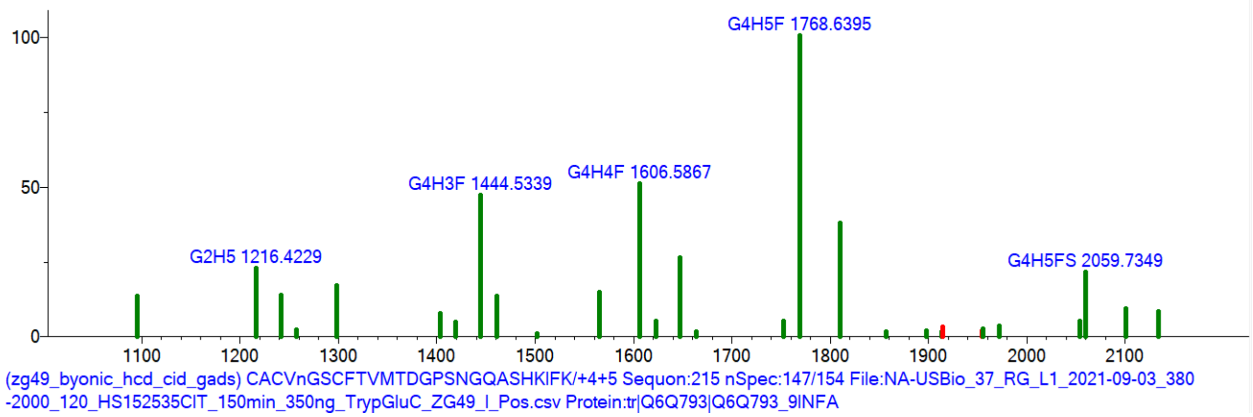

<u>Neuraminidase from strain A/Netherlands/219/2003</u>

Glycosylation site 87

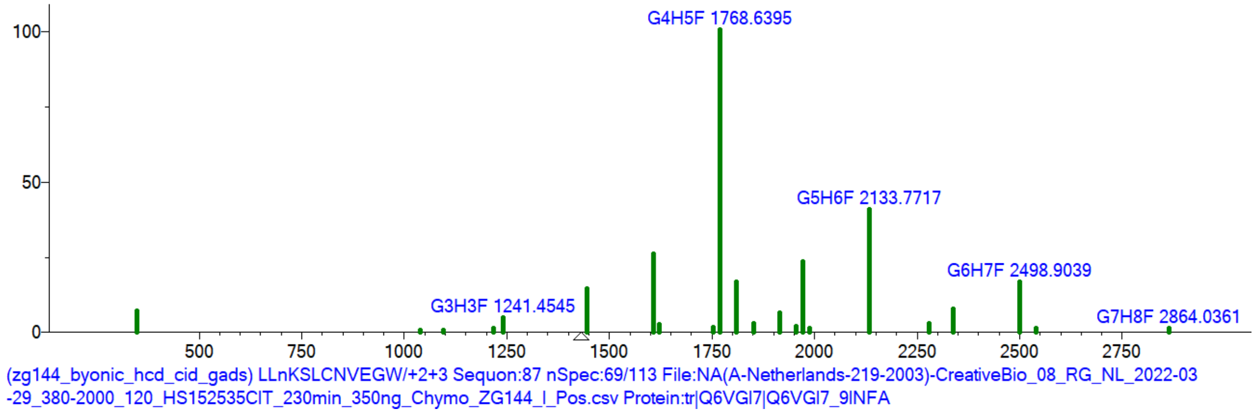

Glycosylation site 145

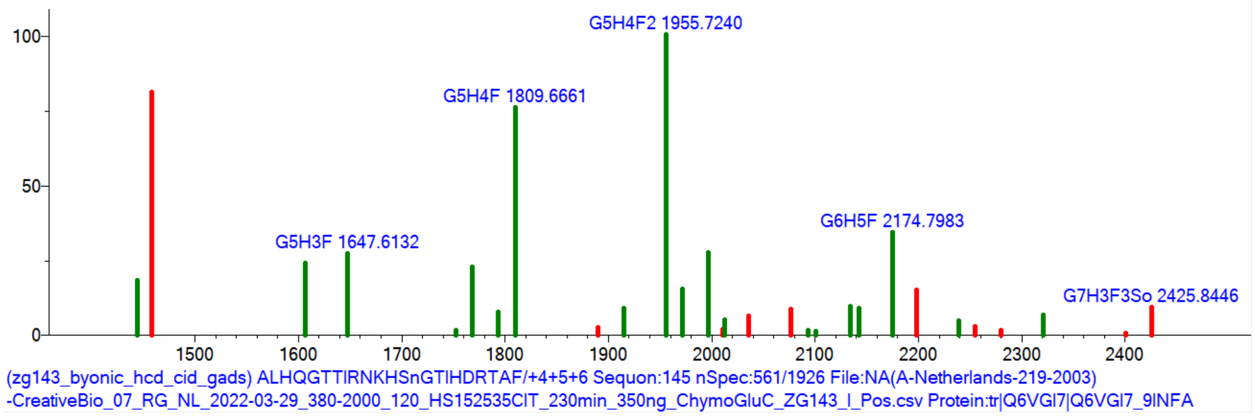

Glycosylation site 180

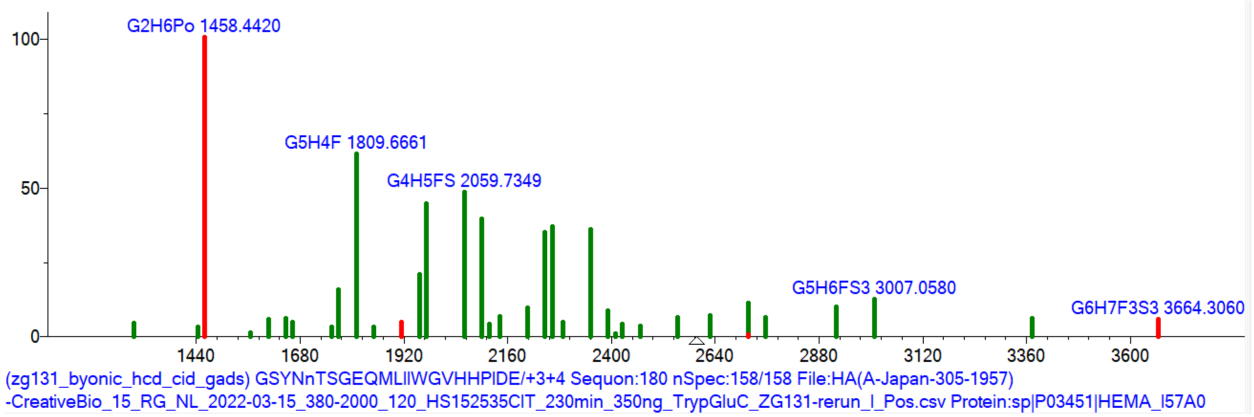

Glycosylation site 200

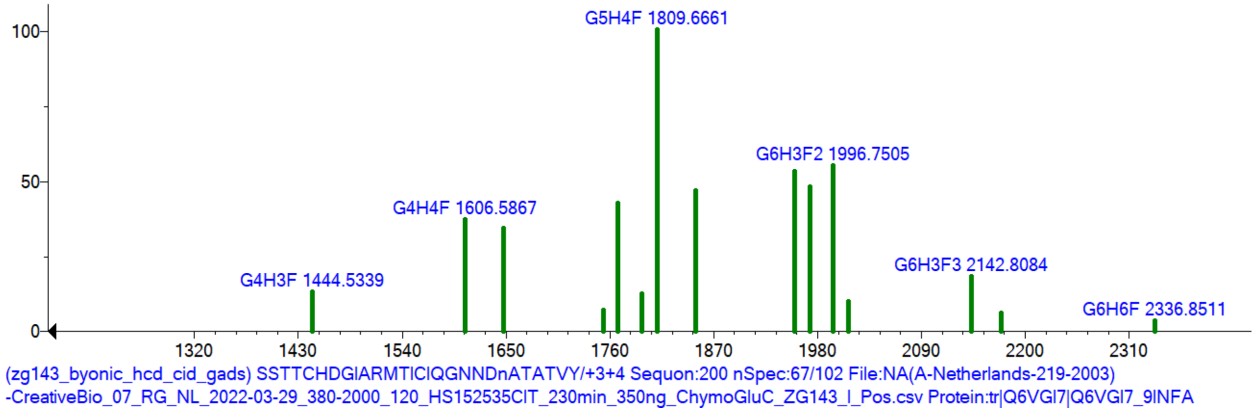

Glycosylation site 234

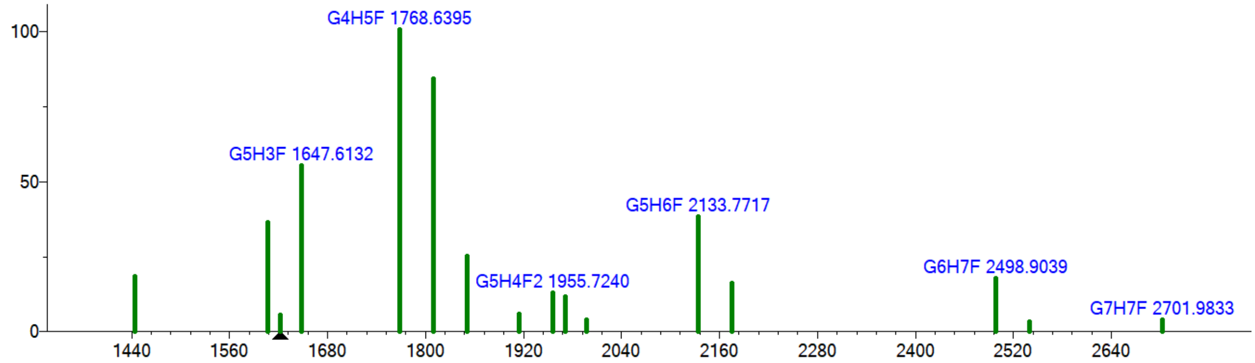

(zg143_byonic_hcd_cid_gads) CVCHnGTCAVVMTD/+2+3 Sequon:234 nSpec:62/96 File:NA(A-Netherlands-219-2003)-CreativeBio_07_RG_NL_2022-03-29_380-2000_120_HS152535CIT_230min_350ng_ChymoGluC_ZG143_I_Pos.csv Protein:tr|Q6VGI7|Q6VGI7_9INFA

Glycosylation site 300

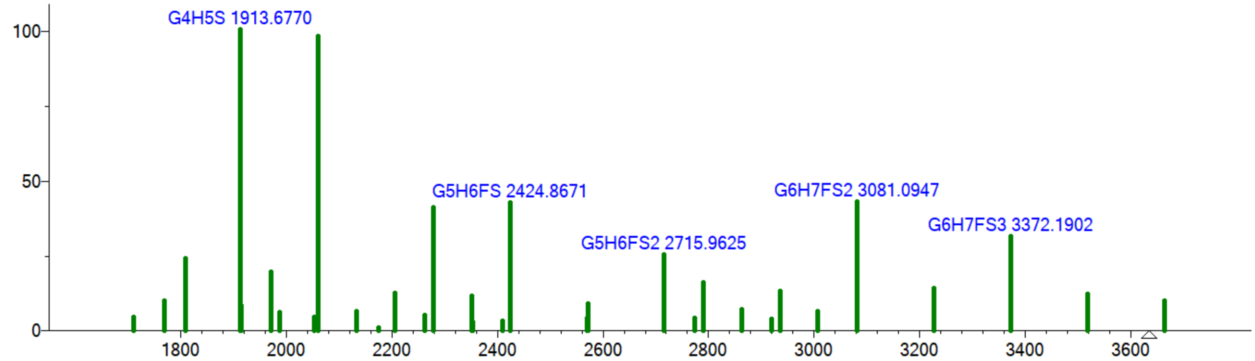

(zg131_byonic_hcd_cid_gads) TKCQTPLGAInTTLPFHNVHPLTIGECPKYVK/+5+6 Sequon:300 nSpec:162/162 File:HA(A-Japan-305-1957)-CreativeBio_15_RG_NL_2022-03-15_380-2000_120_HS152535CIT_230min_350ng_TrypGluC_ZG131-rerun_I_Pos.csv Protein:sp|P03451|HEMA_I57A0

<u>Neuraminidase from strain A/Arizona/13/2008</u>

Glycosylation site 146

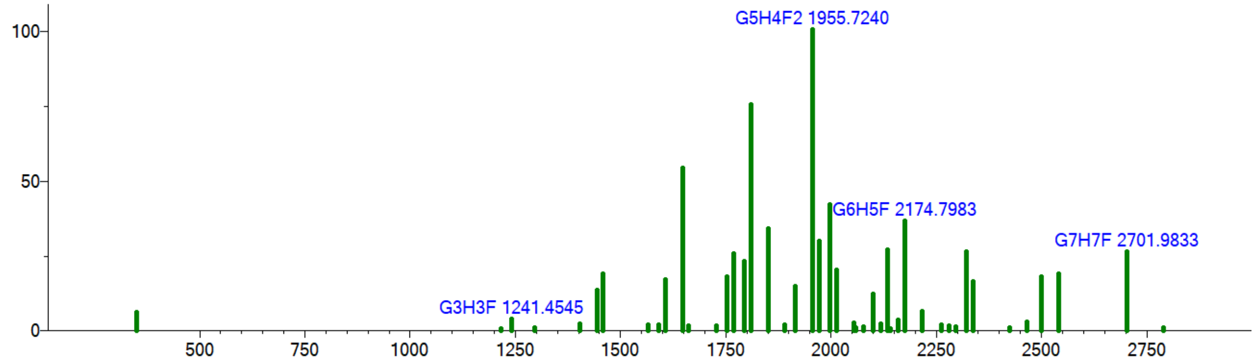

(zg160_byonic_hcd_cid_gads) TFFLTQGALLNDKHSnGTVKDRSPYR/+4+5+6 Sequon:146 nSpec:505/1332 File:NA(A-Arizona-13-2008)-SinoBio_06_RG_NL_2022-04-13_380-2000_120_HS152535CIT_230min_350ng_TrypLysC_ZG160_I_Pos.csv Protein:ACM51249.1

Glycosylation site 235

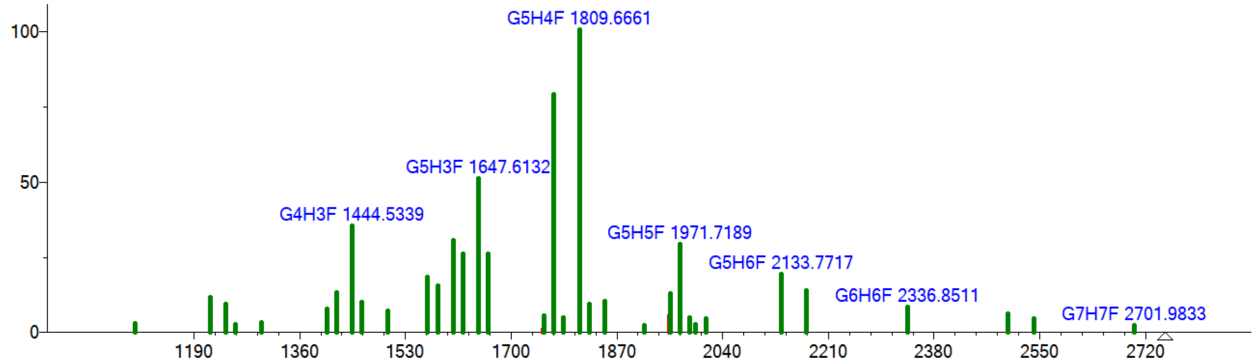

(zg160_byonic_hcd_cid_gads) TQESECVCMnGSCFTIMTDGPSNK/+3+4 Sequon:235 nSpec:251/286 File:NA(A-Arizona-13-2008)-SinoBio_06_RG_NL_2022-04-13_380-2000_120_HS152535CIT_230min_350ng_TrypLysC_ZG160_I_Pos.csv Protein:ACM51249.1

Glycosylation site 434

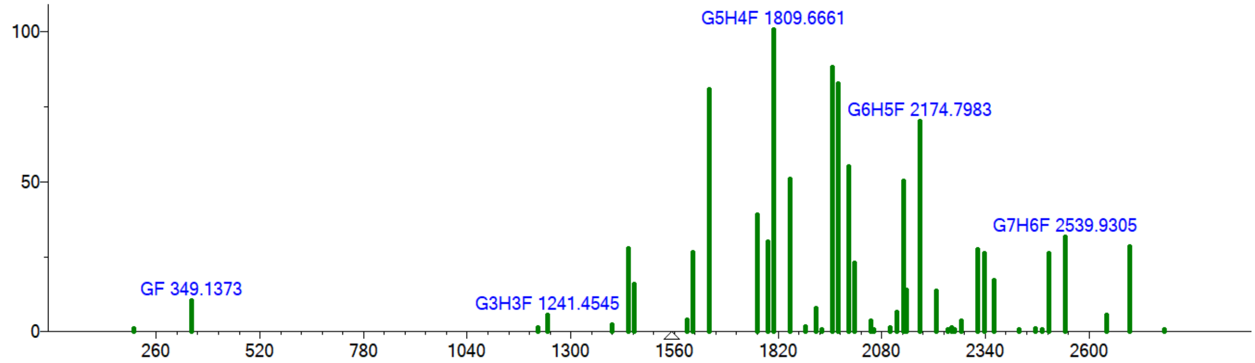

(zg162_byonic_hcd_cid_gads) LVRGLPREnTTIW/+2+3+4 Sequon:434 nSpec:165/222 File:NA(A-Arizona-13-2008)-SinoBio_08_RG_NL_2022-04-13_380-2000_120_HS152535CIT_230min_350ng_ChymoGluC_ZG162_I_Pos.csv Protein:ACM51249.1

Glycosylation site 455

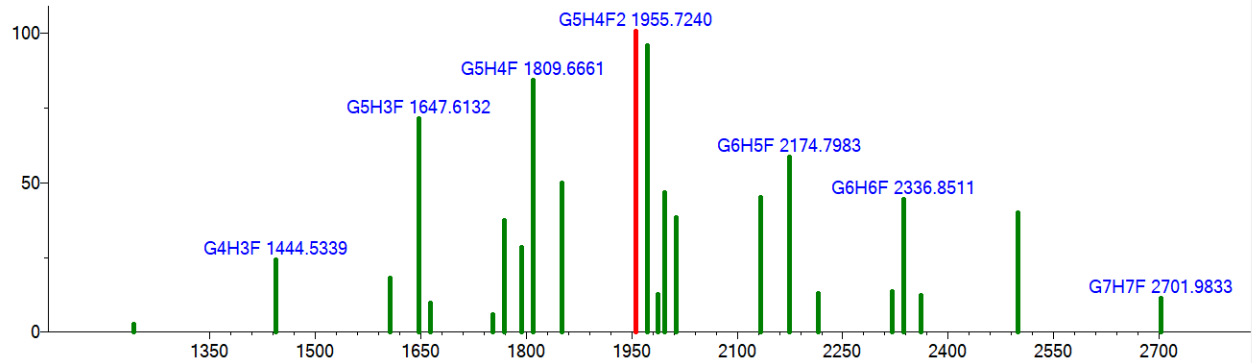

(zg160_byonic_hcd_cid_gads) GLPRENTTIWTSGSSISFCGVNSDTAnWSWPDGAELPFTID/+4+5 Sequon:455 nSpec:106/326 File:NA(A-Arizona-13-2008)-SinoBio_06_RG_NL_2022-04-13_380-2000_120_HS152535CIT_230min_350ng_TrypLysC_ZG160_I_Pos.csv Protein:ACM51249.1
