# Supplementary material for: Variation of Site-Specific Glycosylation Profiles of Recombinant Influenza Glycoproteins
Source: Mol Cell Proteomics. 2024 Aug 10;23(9):100827. doi: 10.1016/j.mcpro.2024.100827 (PMC11417209; doi:10.1016/j.mcpro.2024.100827)
Supplement: Supplemental Document S2 [file mmc2.docx]

Supporting Information for:

Variation of Site-Specific Glycosylation Profiles for Recombinant Influenza Glycoproteins

**Authors:**

Zachary C. Goecker^1,*^; Meghan C. Burke^1^; Concepcion A. Remoroza^1^; Yi Liu^1^; Yuri A. Mirokhin^1^; Sergey L. Sheetlin^1^; Dmitrii V. Tchekhovskoi^1^; Xiaoyu Yang^1^, Stephen E. Stein^1^

^1^Mass Spectrometry Data Center, National Institute of Standards and Technology, 100 Bureau Drive, Gaithersburg, Maryland 20899, United States

*Corresponding Author

**SUPPORTING INFORMATION**

**Page S-1:** Figure S1. Sequence alignment of five hemagglutinin strains

**Page S-2:** Figure S2. Sequence alignment of three neuraminidase strains

**Page S-3:** Table S1. Raw files and their corresponding information

**Page S-4**: Table S2. Supplier information

Figure S3. Inverse similarity score pairwise comparison of all robust glycopeptides identified

**Page S-5**: Figure S4. Plot of similarity score as a function of spectral IDs

**Page S-6**: Figure S5. GADS comparison of replicates with variation in G4H5FS peak

Figure S6. GADS comparison of replicates with variation in G3H3 peak

**Page S-7**: Figure S7. GADS comparison of replicates with variation in G4H4F peak

Figure S8. Figure S6. GADS comparison of different lots with variation in G5 complex peaks

**Page S-8:** Figure S9. GADS comparison of different batches with variation in G4 complex peaks

**Page S-9,10**: Discussion of protein coverage and glycosylation coverage

Figure S10. Glycosylation coverage for the proteomic detection of glycopeptides

**Page S-10,11**: Discussion on the annotation of red GADS peaks

Figure S11. GADS comparison of different proteases with variation in G4 complex peaks

**Page S-12**: Table S3. Number of glycopeptide identifications for each file analyzed

**Page S-13**: Table S4. GADS variation by glycosylation site between three suppliers

**Page S-14:** Figure S12. GADS comparison of similar inter-strain glycosylation sites in protein NA-AZ08

Figure S13. GADS comparison of similar inter-strain glycosylation sites in protein HA-HK97

**Page S-15:** Figure S14. GADS comparison of dissimilar inter-strain glycosylation sites in protein HA-NC99

**Page S-16:** Figure S15. Comparison of glycan distributions from unrelated proteins

**Page S-17:** Figure S16. Comparison of N-glycans between hemagglutinin and SARS-CoV-2 spike protein

--Supplemental document attached to the manuscript separately--

Document S1. **Representative GADS for each glycosylation site in all proteins measured**

CLUSTAL O(1.2.4) multiple sequence alignment

HA A/Hong Kong/485197/2014 (H3N2) MKTIIALSYILCLVFAQKIPGNDNSTATLCLGHHAVPNGTIVKTITNDRIEVTNATELVQ 60

HA A/California/04/2009 (H1N1) MKA--ILVVLLYTF-------ATANADTLCIGYHANNSTDTVDTVLEKNVTVTHSVNLLE 51

HA A/New Caledonia/20/1999 (H1N1) MKA--KLLVLLCTF-------TATYADTICIGYHANNSTDTVDTVLEKNVTVTHSVNLLE 51

HA A/Japan/305/1957 (H2N2) --M--AIIYLILLF-------TAVRGDQICIGYHANNSTEKVDTNLERNVTVTHAKDILE 49

HA A/Hong Kong/483/1997 (H5N1) -ME--KIVLLLATV-------SLVKSDQICIGYHANNSTEQVDTIMEKNVTVTHAQDILE 50

: :: . :*:*:** . *.* : .: **:: ::::

HA A/Hong Kong/485197/2014 (H3N2) NSSIGEICDSPH-QILDGENCTLIDALLGDPQCDGFQN-KKWDLFVERS-KAYSNCYPYD 117

HA A/California/04/2009 (H1N1) DKHNGKLCKLRGVAPLHLGKCNIAGWILGNPECESLSTASSWSYIVETPSSDNGTCYPGD 111

HA A/New Caledonia/20/1999 (H1N1) DSHNGKLCLLKGIAPLQLGNCSVAGWILGNPECELLISKESWSYIVETPNPENGTCYPGY 111

HA A/Japan/305/1957 (H2N2) KTHNGKLCKLNGIPPLELGDCSIAGWLLGNPECDRLLSVPEWSYIMEKENPRDGLCYPGS 109

HA A/Hong Kong/483/1997 (H5N1) RTHNGKLCDLNGVKPLILRDCSVAGWLLGNPMCDEFINVPEWSYIVEKASPANDLCYPGN 110

. *::* * .*.: . :**:* *: : . .*. ::* . ***

HA A/Hong Kong/485197/2014 (H3N2) VPDYASLRSLVATSGTLE---FNNESFNWTGVTQ-NGTSSACIRRSSSSFFSRLNWLTHL 173

HA A/California/04/2009 (H1N1) FIDYEELREQLSSVSSFERFEIFPKTSSWPNHDSNKGVTAACPHAGAKSFYKNLIWLVKK 171

HA A/New Caledonia/20/1999 (H1N1) FADYEELREQLSSVSSFERFEIFPKESSWPNHTV-TGVSASCSHNGKSSFYRNLLWLTGK 170

HA A/Japan/305/1957 (H2N2) FNDYEELKHLLSSVKHFEKVKILPK-DRWTQHTTT-GGSRACAVSGNPSFFRNMVWLTKE 167

HA A/Hong Kong/483/1997 (H5N1) FNDYEELKHLLSRINHFEKIQIIPK-SSWSNHDASSGVSSACPYLGKSSFFRNVVWLIKK 169

. ** .*: :: :* : : * * : :* . **: .: **

HA A/Hong Kong/485197/2014 (H3N2) NYTYPALNVTMPNNEQFDKLYIWGVHHPGTDKDQIFLYAQSSGRITVSTKRSQQAVIPNI 233

HA A/California/04/2009 (H1N1) GNSYPKLSKSYINDKGKEVLVLWGIHHPSTSADQQSLYQNADTYVFVGSSRYSKKFKPEI 231

HA A/New Caledonia/20/1999 (H1N1) NGLYPNLSKSYVNNKEKEVLVLWGVHHPPNIGNQRALYHTENAYVSVVSSHYSRRFTPEI 230

HA A/Japan/305/1957 (H2N2) GSDYPVAKGSYNNTSGEQMLIIWGVHHPIDETEQRTLYQNVGTYVSVGTSTLNKRSTPEI 227

HA A/Hong Kong/483/1997 (H5N1) NSTYPTIKRSYNNTNQEDLLVLWGIHHPNDAAEQTKLYQNPTTYISVGTSTLNQRLVPEI 229

. ** . : * . : * :**:*** :* ** : * :. .: *:*

HA A/Hong Kong/485197/2014 (H3N2) GSRPRIRDIPSRISIYWTIVKPGDILLINSTGNLIAPRGYFKI-RSGKSSIMRSDAPIGK 292

HA A/California/04/2009 (H1N1) AIRPKVRDQEGRMNYYWTLVEPGDKITFEATGNLVVPRYAFAMERNAGSGIIISDTPVHD 291

HA A/New Caledonia/20/1999 (H1N1) AKRPKVRDQEGRINYYWTLLEPGDTIIFEANGNLIAPWYAFALSRGFGSGIITSNAPMDE 290

HA A/Japan/305/1957 (H2N2) ATRPKVNGQGGRMEFSWTLLDMWDTINFESTGNLIAPEYGFKISKRGSSGIMKTEGTLEN 287

HA A/Hong Kong/483/1997 (H5N1) ATRPKVNGQSGRIEFFWTILKPNDAINFESNGNFIAPEYAYKIVKKGDSTIMKSELEYGN 289

. **::.. .*:. **::. * : :::.**::.* : : : * *: :: .

HA A/Hong Kong/485197/2014 (H3N2) CKSECITPNGSIPNDKPFQNVNRITYGACPRYVKHSTLKLATGMRNVPEKQ----TRGIF 348

HA A/California/04/2009 (H1N1) CNTTCQTPKGAINTSLPFQNIHPITIGKCPKYVKSTKLRLATGLRNIPSIQ----SRGLF 347

HA A/New Caledonia/20/1999 (H1N1) CDAKCQTPQGAINSSLPFQNVHPVTIGECPKYVRSAKLRMVTGLRNIPSIQ----SRGLF 346

HA A/Japan/305/1957 (H2N2) CETKCQTPLGAINTTLPFHNVHPLTIGECPKYVKSEKLVLATGLRNVPQIE----SRGLF 343

HA A/Hong Kong/483/1997 (H5N1) CNTKCQTPMGAINSSMPFHNIHPLTIGECPKYVKSNRLVLATGLRNAPQRERRRKKRGLF 349

*.: * ** *:* . **:*:: :* * **:**: * :.**:** *. : .**:*

HA A/Hong Kong/485197/2014 (H3N2) GAIAGFIENGWEGMVDGWYGFRHQNSEGRGQAADLKSTQAAIDQINGKLNRLIGKTNEKF 408

HA A/California/04/2009 (H1N1) GAIAGFIEGGWTGMVDGWYGYHHQNEQGSGYAADLKSTQNAIDEITNKVNSVIEKMNTQF 407

HA A/New Caledonia/20/1999 (H1N1) GAIAGFIEGGWTGMVDGWYGYHHQNEQGSGYAADQKSTQNAINGITNKVNSVIEKMNTQF 406

HA A/Japan/305/1957 (H2N2) GAIAGFIEGGWQGMVDGWYGYHHSNDQGSGYAADKESTQKAFDGITNKVNSVIEKMNTQF 403

HA A/Hong Kong/483/1997 (H5N1) GAIAGFIEGGWQGMVDGWYGYHHSNEQGSGYAADQESTQKAIDGVTNKVNSIIDKMNTQF 409

********.** ********::*.*.:* * *** :*** *:: :..*:* :* * * :*

HA A/Hong Kong/485197/2014 (H3N2) HQIEKEFSEVEGRIQDLEKYVEDTKIDLWSYNAELLVALENQHTIDLTDSEMNKLFEKTK 468

HA A/California/04/2009 (H1N1) TAVGKEFNHLEKRIENLNKKVDDGFLDIWTYNAELLVLLENERTLDYHDSNVKNLYEKVR 467

HA A/New Caledonia/20/1999 (H1N1) TAVGKEFNKLERRMENLNKKVDDGFLDIWTYNAELLVLLENERTLDFHDSNVKNLYEKVK 466

HA A/Japan/305/1957 (H2N2) EAVGKEFGNLERRLENLNKRMEDGFLDVWTYNAELLVLMENERTLDFHDSNVKNLYDKVR 463

HA A/Hong Kong/483/1997 (H5N1) EAVGREFNNLERRIENLNKKMEDGFLDVWTYNAELLVLMENERTLDFHDSNVKNLYDKVR 469

: :**..:* *:::*:* ::* :*:*:******* :**::*:* **::::*::*.:

HA A/Hong Kong/485197/2014 (H3N2) KQLRENAEDMGNGCFKIYHKCDNACIGSIRNGTYDHNVYRDEALNNRFQIKGVELKSGYK 528

HA A/California/04/2009 (H1N1) SQLKNNAKEIGNGCFEFYHKCDNTCMESVKNGTYDYPKYSEEAKLNREEIDGVKLESTRI 527

HA A/New Caledonia/20/1999 (H1N1) SQLKNNAKEIGNGCFEFYHKCNNECMESVKNGTYDYPKYSEESKLNREKIDGVKLESMGV 526

HA A/Japan/305/1957 (H2N2) MQLRDNVKELGNGCFEFYHKCDDECMNSVKNGTYDYPKYEEESKLNRNEIKGVKLSSMGV 523

HA A/Hong Kong/483/1997 (H5N1) LQLRDNAKELGNGCFEFYHKCDNECMESVKNGTYDYPQYSEEARLNREEISGVKLESMGT 529

**::*.:::*****::****:: *: *::*****: * :*: ** :*.**:*.*

HA A/Hong Kong/485197/2014 (H3N2) DWILWI-SFAISCFLLCVALLGFIMWACQKGNIRCNICI 566

HA A/California/04/2009 (H1N1) YQILAIYSTVASSLVLVVSLGAISFWMCSNGSLQCRICI 566

HA A/New Caledonia/20/1999 (H1N1) YQILAIYSTVASSLVLLVSLGAISFWMCSNGSLQCRICI 565

HA A/Japan/305/1957 (H2N2) YQILAIYATVAGSLSLAIMMAGISFWMCSNGSLQCRICI 562

HA A/Hong Kong/483/1997 (H5N1) YQILSLYSTVASSLALAIMVAGLSLWMCSNGSLQCRICI 568

** : : . ..: * : : .: :* *.:*.::*.***

Figure S1. **Sequence alignment of five hemagglutinin strains**

Protein sequences from the five strains of hemagglutinin were processed by the Clustal Omega program^1^ to assess position and overlap. Red boxes indicate glycosylation sites without sequence overlap with other strains, whereas green boxes indicate overlap with other strains.

CLUSTAL O(1.2.4) multiple sequence alignment

NA A/Netherlands/219/2003 (H7N7) MNPNQKLFALSGVAIALSVLNLLIGISNVGLNVSLHLKEKGPKQEENLT-CTTINQNNTT 59

NA A/Arizona/13/2008 (H1N1) MNPNQKIITIGSISIAIGIISLMLQIGSIISIWASHSIQTGSQNSTGICNQRIITYENST 60

NA A/Thailand/1(KAN-1)/2004 (H5N1) MNPNKKIITIGSICMVTGMVSLMLQIGNLISIWVSHSIHTGNQHKAE------------- 47

****:*::::..:.:. .::.*:: *..: * ..* ::.

NA A/Netherlands/219/2003 (H7N7) VVENTYVNNTTIITKGTDLKTPSYLLLNKSLCNVEGWVVIAKDNAVRFGESEQIIVTREP 119

NA A/Arizona/13/2008 (H1N1) WVNHTYVNINNTNVVAGEDKTSVTLVGNSSLCSISGWAIYTKDNSIRIGSKGDVFVIREP 120

NA A/Thailand/1(KAN-1)/2004 (H5N1) -------PISNTNFLTEKAVASVKLAGNSSLCPINGWAVYSKDNSIRIGSKGDVFVIREP 100

.. . : * *.*** :.**.: :***::*:*.. :::* ***

NA A/Netherlands/219/2003 (H7N7) YVSCDPTGCKMYALHQGTTIRNKHSNGTIHDRTAFRGLISTPLGTPPTVSNSDFMCVGWS 179

NA A/Arizona/13/2008 (H1N1) FISCSHLECRTFFLTQGALLNDKHSNGTVKDRSPYRALMSCPLGEAPSPYNSKFESVAWS 180

NA A/Thailand/1(KAN-1)/2004 (H5N1) FISCSHLECRTFFLTQGALLNDKHSNGTVKDRSPHRTLMSCPVGEAPSPYNSRFESVAWS 160

::**. *: : * **: :.:******::**: .* *:* *:* *: ** * .*.**

NA A/Netherlands/219/2003 (H7N7) STTCHDGIARMTICIQGNNDNATATVYYNRRLTTTIKTWARNILRTQESECVCHNGTCAV 239

NA A/Arizona/13/2008 (H1N1) ASACHDGMGWLTIGISGPDNGAVAVLKYNGIITGTIKSWKKQILRTQESECVCMNGSCFT 240

NA A/Thailand/1(KAN-1)/2004 (H5N1) ASACHDGTSWLTIGISGPDNGAVAVLKYNGIITDTIKSWRNNILRTQESECACVNGSCFT 220

:::**** . :** *.* ::.*.*.: ** :* ***:* .:*********.* **:* .

NA A/Netherlands/219/2003 (H7N7) VMTDGSASSQAYTKVMYFHKGLVVKEEELRGSARHIEECSCYGHNQKVTCVCRDNWQGAN 299

NA A/Arizona/13/2008 (H1N1) IMTDGPSNKAASYKIFKIEKGKVTKSIELNAPNFYYEECSCYPDTGIVMCVCRDNWHGSN 300

NA A/Thailand/1(KAN-1)/2004 (H5N1) VMTDGPSNGQASHKIFKMEKGKVVKSVELDAPNYHYEECSCYPDAGEITCVCRDNWHGSN 280

:**** :. * *:: :.** *.*. ** . : ****** . : *******:*:*

NA A/Netherlands/219/2003 (H7N7) RPIIEIDMSTLEHTSRYVCTGILTDTSRPGDKSSGDCSNPITGSPGVPGVKGFGFLNGDN 359

NA A/Arizona/13/2008 (H1N1) RPWVSFNQ-NLDYQIGYICSGVFGDNPRPEDGE-GSCN--PVTVDGANGVKGFSYKYGNG 356

NA A/Thailand/1(KAN-1)/2004 (H5N1) RPWVSFNQ-NLEYQIGYICSGVFGDNPRPNDGT-GSCG--PVSSNGAYGVKGFSFKYGNG 336

** :.:: .*:: *:*:*:: *. ** * *.*. . *. *****.: *:.

NA A/Netherlands/219/2003 (H7N7) TWLGRTISPRSRSGFEMLKIPNAGTDPNSRIAERQEIVDNNNWSGYSGSFIDYWN-DNSE 418

NA A/Arizona/13/2008 (H1N1) VWIGRTKSNRLRKGFEMIWDPNGWTNTDSDFSVKQDVVAITDWSGYSGSFVQHPELTGLD 416

NA A/Thailand/1(KAN-1)/2004 (H5N1) VWIGRTKSTNSRSGFEMIWDPNGWTETDSSFSVKQDIVAITDWSGYSGSFVQHPELTGLD 396

.*:*** * . *.****: **. *: :* :: :*::* .:********::: : . :

NA A/Netherlands/219/2003 (H7N7) CYNPCFYVELIRGRPEEAKYVWWASNSLIALCGSPFPVGSGSFPDGAQIQYFS-- 471

NA A/Arizona/13/2008 (H1N1) CIRPCFWVELVRGLPRENTTIW-TSGSSISFCGVNSDTANWSWPDGAELPFTIDK 470

NA A/Thailand/1(KAN-1)/2004 (H5N1) CIRPCFWVELIRGRPKE-STIW-TSGSSISFCGVNSDTVGWSWPDGAELPFTIDK 449

* .***:***:** *.* . :* :*.* *::** . . *:****:: :

Figure S2. **Sequence alignment of three neuraminidase strains**

Protein sequences from the three strains of neuraminidase were input into the Clustal Omega program^1^ to assess position and any potential overlap. Red boxes indicate glycosylation sites that do not have sequence overlap with another strain, whereas green boxes indicate overlap with other strains.

Reference:

1. Madeira, F.; Park, Y. M.; Lee, J.; Buso, N.; Gur, T.; Madhusoodanan, N.; Basutkar, P.; Tivey, A. R. N.; Potter, S. C.; Finn, R. D.; Lopez, R. The EMBL-EBI search and sequence analysis tools APIs in 2019. *Nucleic Acids Res* **2019**, *47* (W1), W636-W641. DOI: 10.1093/nar/gkz268 From NLM Medline.

Table S1. **List of raw files uploaded to repository and corresponding information**


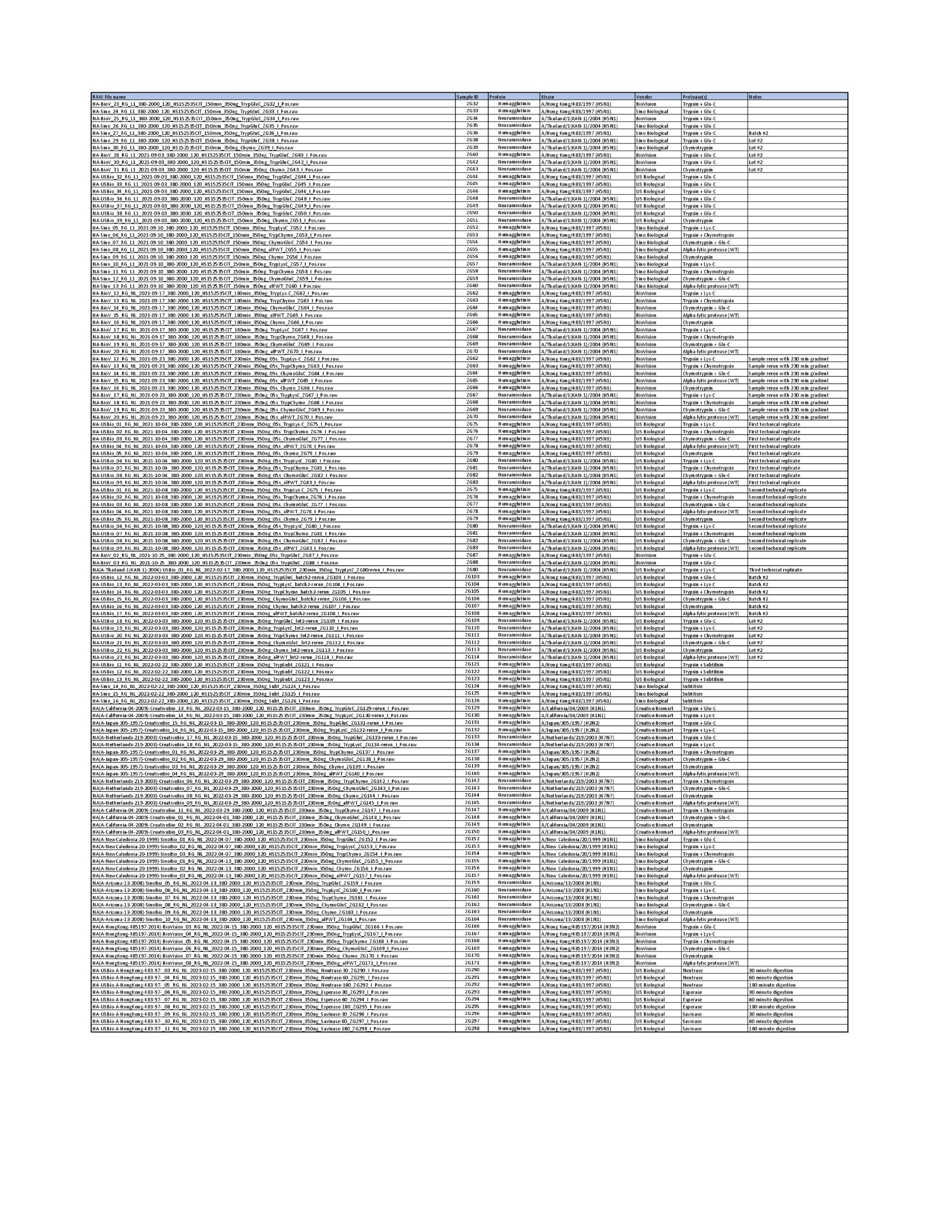


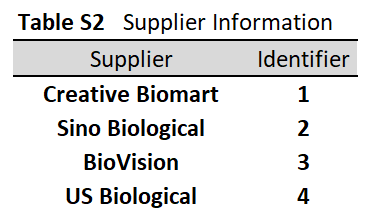


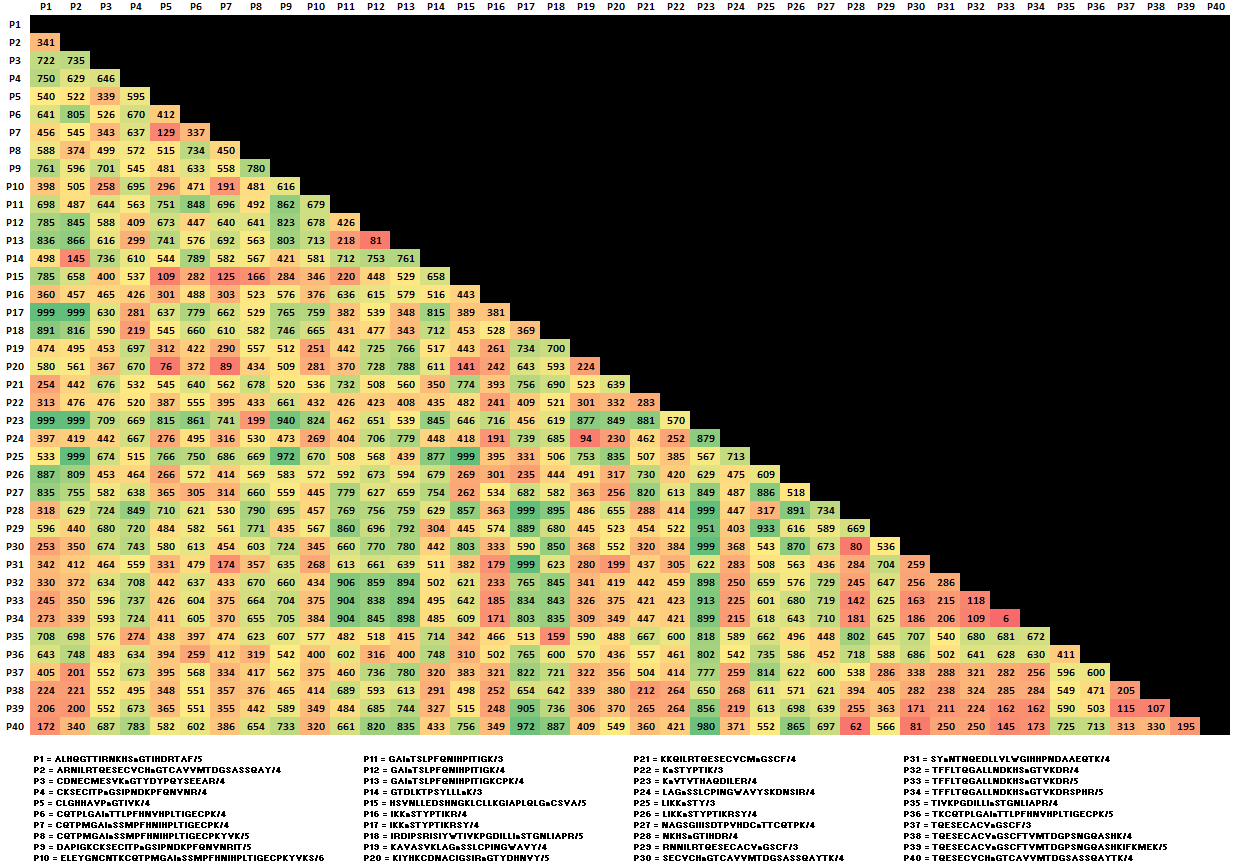


Figure S3. **Pairwise** **comparisons of all good quality GADS**

All GADS from recombinant HA and NA with numbers of identified glycopeptides (nSpec > 100) were compared, excluding replicates. These scores are 1 - dot product, which transform high similarity to low numerical value, enabling hierarchical clustering of similar data. Note than a 999 indicate no peaks in common. A threshold similarity score of 500 was used for clustering into groups. Four such GADS classes resulted. These classes are employed in Figure 4 of the manuscript.


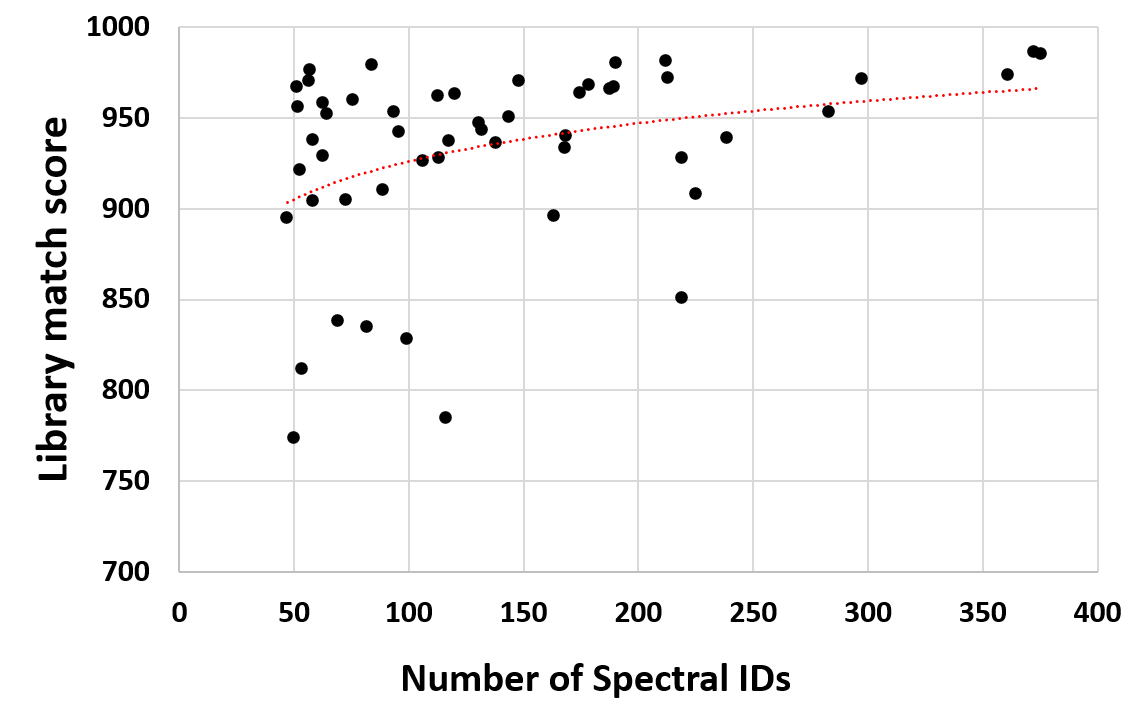


Figure S4. **Similarity score versus of numbers of glycopeptide IDs**

Similarities of GADS for the same peptide sequence for injection replicates versus numbers of spectral IDs. GADS reproducibility increases with increasing numbers of spectral identifications. Low match score outliers are primarily due to missing glycans as described in Table 3 of the manuscript.


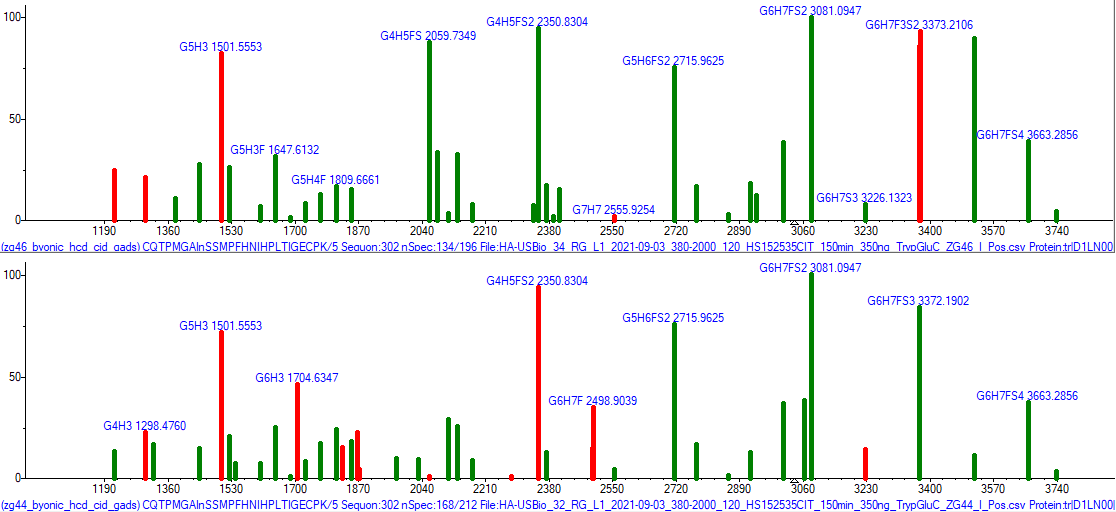


Figure S5. **GADS comparison of two digestion replicates showing variation in G4H5FS peak**

GADS compared are both from protein HA-HK97 from supplier 4 at glycosylation site 302 for two equivalent digests performed with trypsin + GluC. GADS are from the same peptide sequence and same charge state.


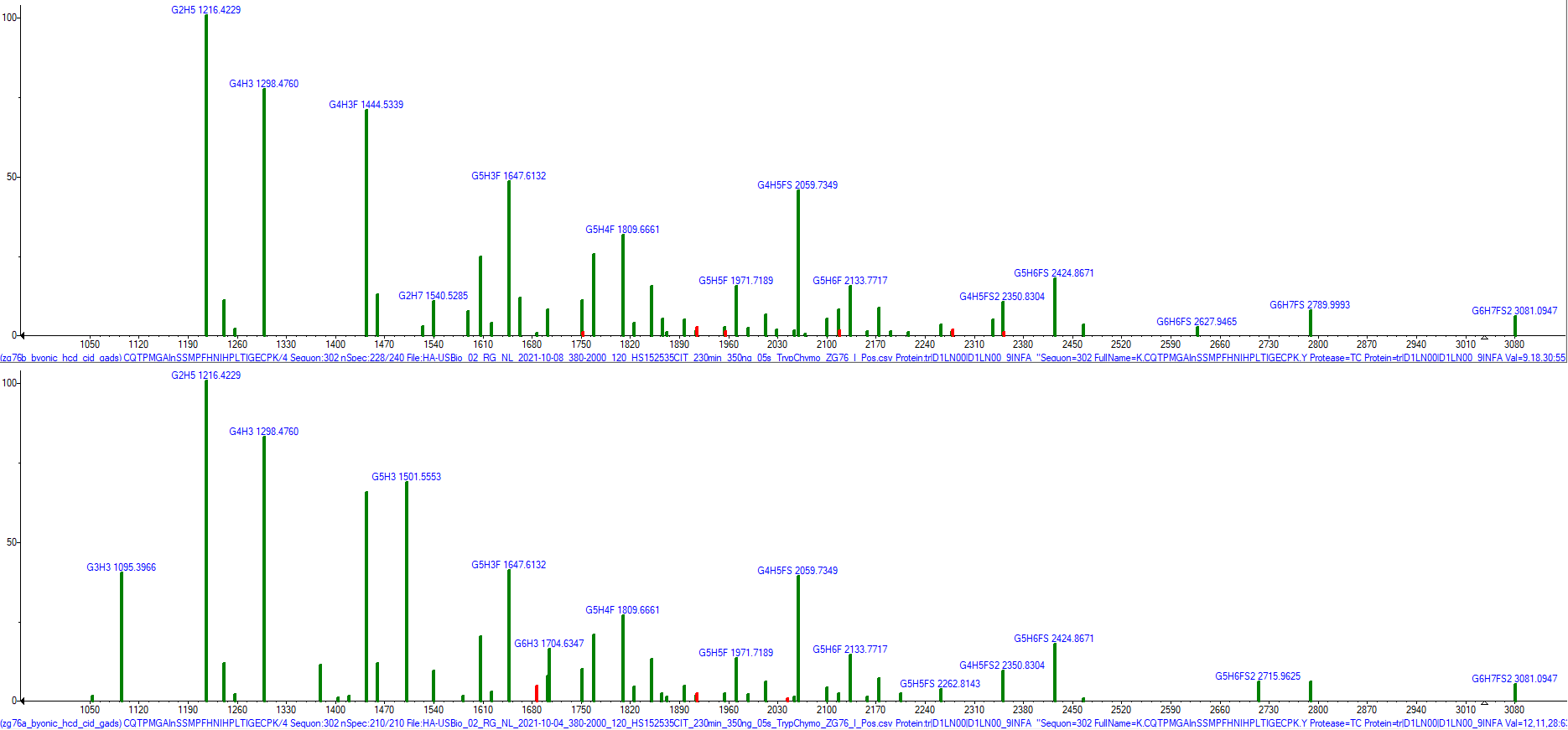


Figure S6. **GADS comparison of two injection replicates showing variation in G3H3 peak**

Illustrative GADS from protein HA-HK97 from supplier 4 at glycosylation site 302 showing missing peak in upper GADS probably due to sampling problem. Note that GADS are from repeat injections of the same sample (ZG76) which was digested using trypsin + chymotrypsin. GADS are from the same peptide sequence and same charge state.


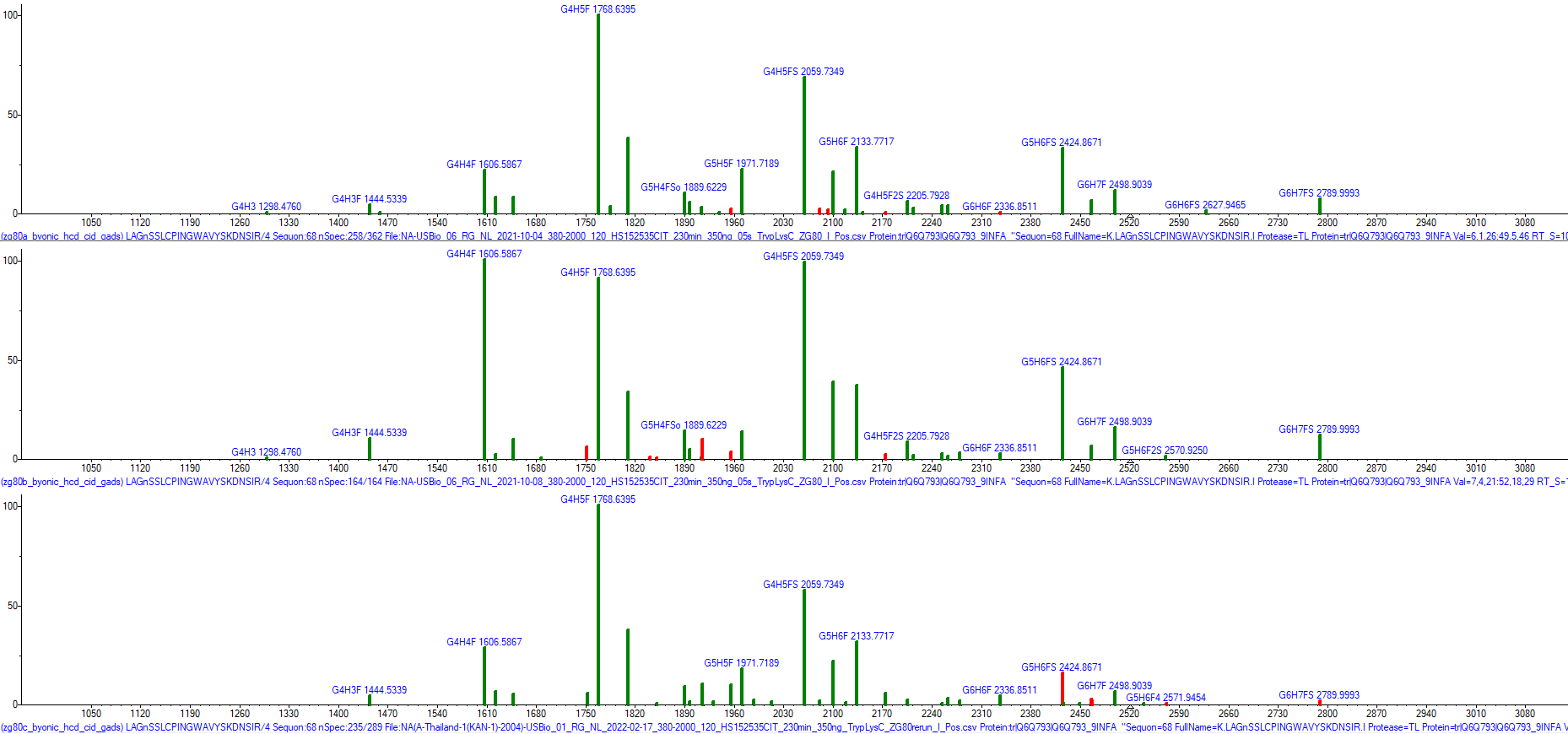


Figure S7. **GADS comparison of three injection replicates showing variations in G4H4F peak**

GADS compared are from protein NA-TH04 from supplier 4 at glycosylation site 68. Note that GADS are from repeat injections of the same sample (ZG80) which was digested using trypsin + Lys-C. GADS are from the same peptide sequence and same charge state. Sometimes abundances vary due to difficulties in extraction of XIC abundances in complex chromatogram.


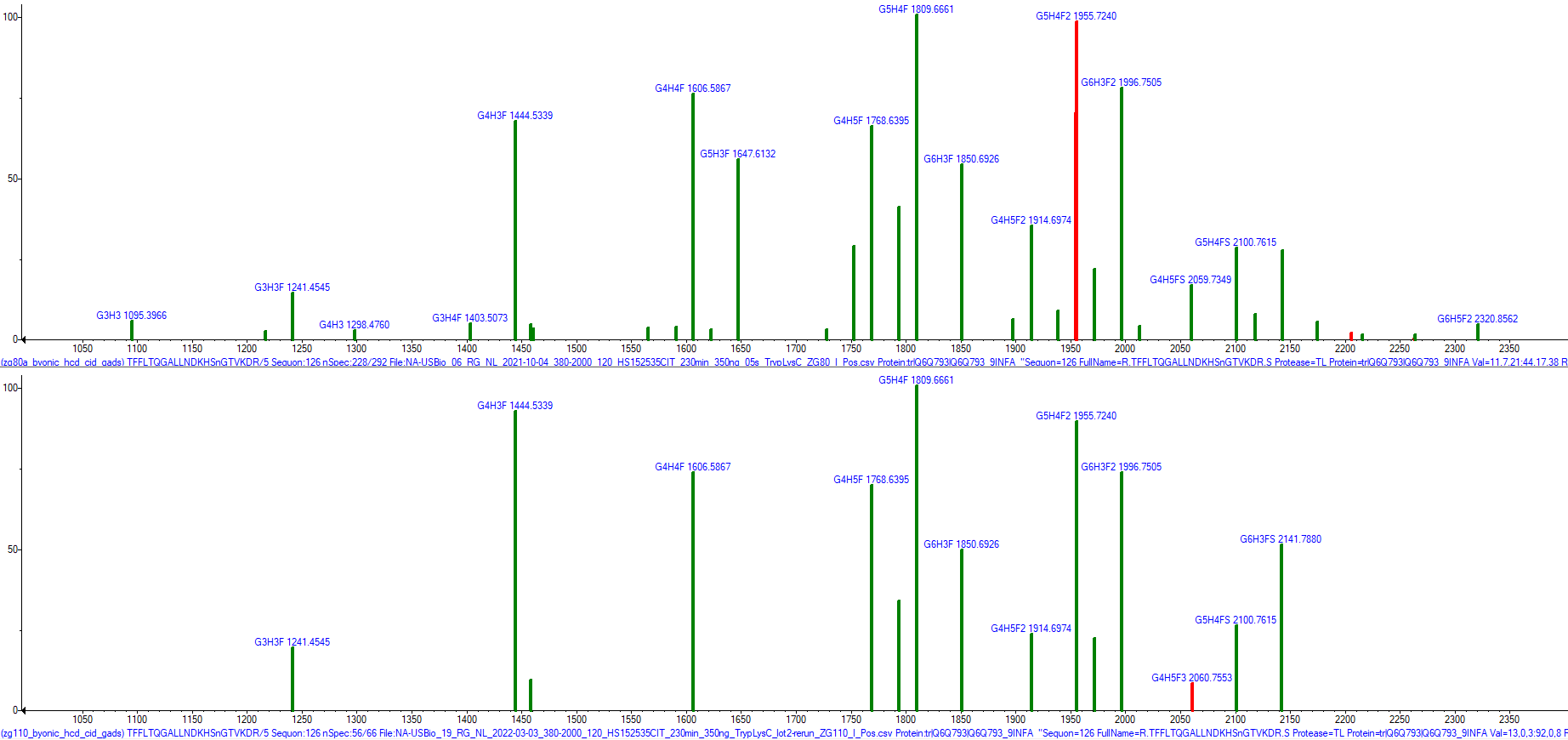


Figure S8. **GADS comparison of two different lots showing variation in G5 complex peaks**

GADS compared are from protein NA-TH04 from supplier 4 at glycosylation site 126. Both digests used trypsin + Lys-C. GADS are from the same peptide sequence and same charge state. Illustration of uncertainty in identification. Likely that ID was missed in lower GADS.


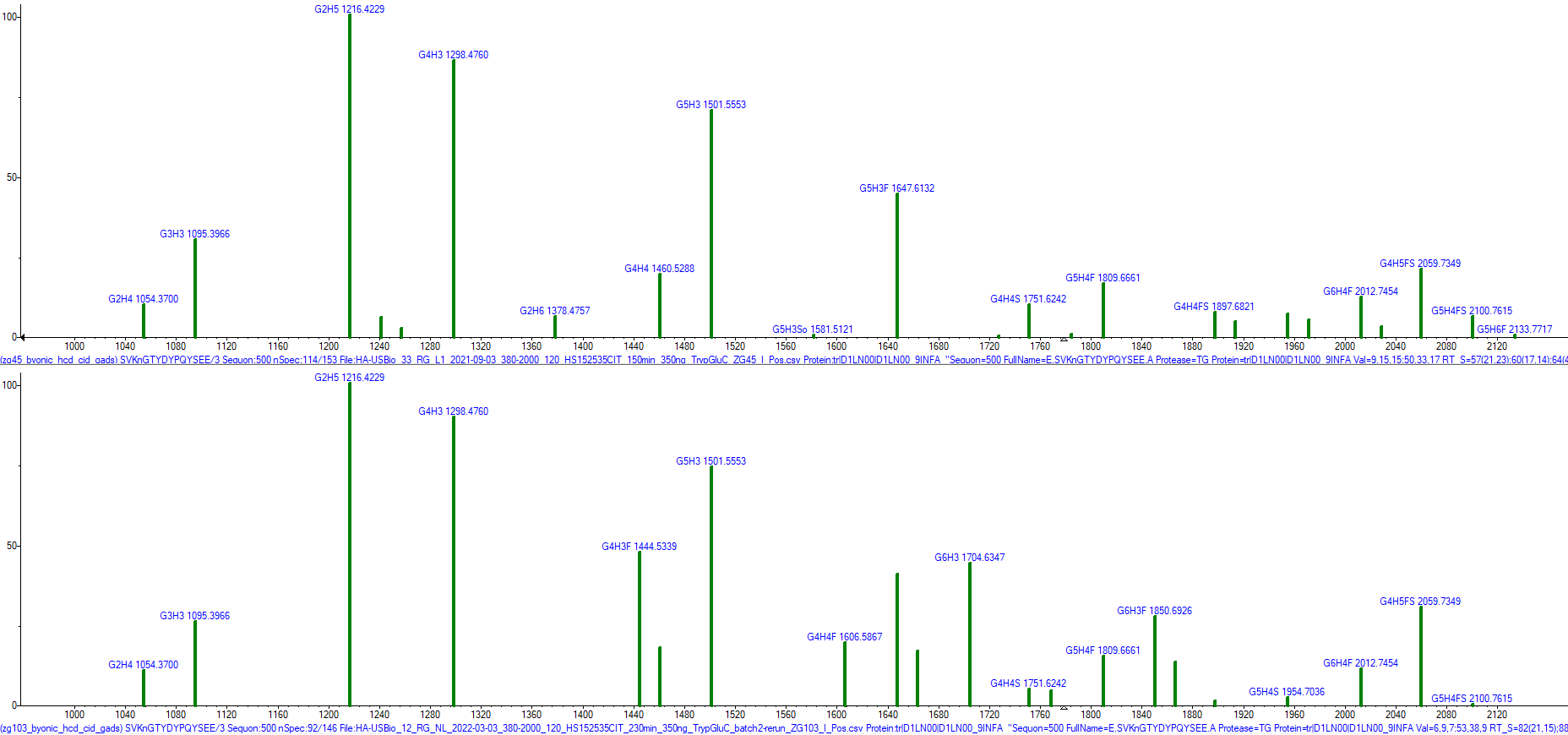


Figure S9. **GADS comparison of two different batches showing variation in G4 complex peaks**

GADS compared are from protein HA-HK97 from supplier 4 at glycosylation site 500. Both digests used trypsin + Glu-C. GADS are from the same peptide sequence and same charge state.

*Glycosylation site coverage comparison*

Each glycoprotein that was analyzed was digested using a series of six protease combinations as mentioned in the methods section. These proteases were used to improve sequence coverage, to detect more glycopeptides add confidence to GADS. However, some glycosylation sites were hardly detected, and others were detected in high abundance regardless of the diversity of proteases used. This high degree of variation occurs primarily when glycosylation sites are in regions of the protein where efficient formation of a suitable peptide containing that site is not possible. Here, we report numbers of glycopeptide identifications at each expected N-glycosylated site for the eight proteins examined, including those obtained from different sources (Figure S8). This is intended to be only illustrative since such details will depend on many factors.

Results here indicate that NA-NL03 yielded the best coverage, with all glycosylation sites detected and most being detected with tens of thousands of identifications. All three vendors for NA-TH04 produced a similar amount of glycopeptide identifications. However, this was not the case for HA-HK97, where Sino Biological produced the highest number of glycopeptide IDs compared to US Biological and BioVision. The protein that had the lowest number of IDs and therefore lowest protein coverage was HA-NC99, where one glycosylation site has no peptide identifications and the first two adjacent glycosylation sites hardly have any identifications. Note that peptides containing more than one glycosylation site are included in Figure S8, since it is primarily intended to illustrate variations in depth of coverage (spectral counts) at each glycosylated site. Across the board, glycosylation sites at the end of the protein such as site 557 in HA-NC99 and HA-CA09, site 554 in HA-JP57, and site 560 in HA-HK97 had few identifications. While not as pronounced, a similar observation was made at the N terminus of the protein where fewer identifications are measured, such as with sites 25 and 26 of HA-JP57, site 44 of NA-AZ08, and sites 27 and 28 of HA-NC99.


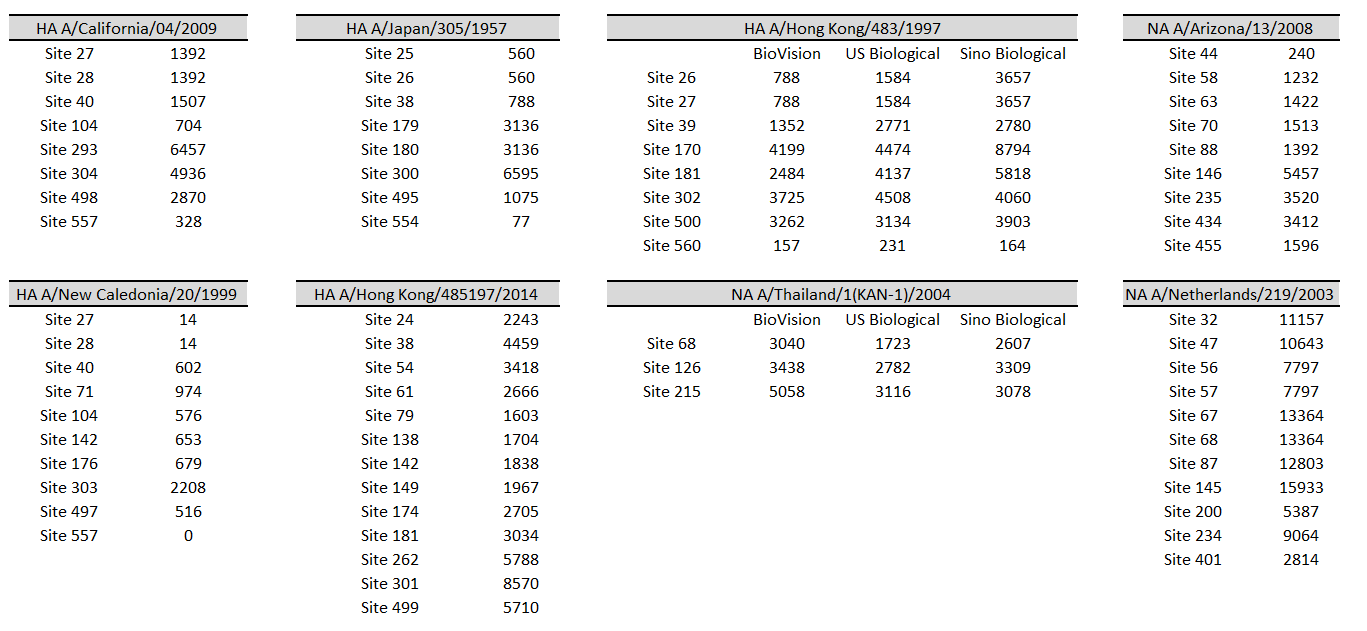


Figure S10. **Glycosylation site coverage based on numbers of identified glycopeptides**

Tables with the number of MS^2^ IDs (spectral counts) corresponding to each site in all proteins analyzed.

*Annotation of red peaks*

The process of glycopeptide identification employed a number of factors to indicate suspect identifications. When problematic identifications were observed, peaks were marked red with details of the problem given in the text for each peak in the GADS. Based on reproducibility studies, most appear to be correct, but should be used with caution. The classes of problems are: (1) A glycopeptide is outside the expected retention time range, set by the program based on coeluting peptides having the same number sialyl groups. (2) A glycopeptide MS2 spectrum and its contingent ion-trap spectrum is missing the Y1 ion. (3) A sialylated glycopeptide has no sialylated oxonium ion. (4) A non-sialylated peptide has a significant (>10%) sialyl oxonium ion (small oxonium ions could arise from co-eluting glycopeptides). (5) A glycopeptide MS2 spectrum had no fucosylated Y ions for glycans with more than one fucose. (6) A possible misidentification of one sialic acid with two fucose sugars (approximately 1 Da higher) due to a missing monoisotopic ion that is inconsistent with retention time. (7) A phosphorylated glycopeptide spectrum that has no phosphorylation oxonium ions. (8) A prominent MS1 peak with relatively few identifications. (9) An oxidized methionine is identified, no expected loss of 64 Da is observed among the Y ions. (10) Sodiation is identified, but there no sodiation is observed in product oxonium ions. (11) Multiple charge states are detected for a glycopeptide, but at significantly different retention times. Many of these problems are rare but would increase in number if lower thresholds for identification were used. They are also limited by the requirement that reported GADS contain a least two different confidently identified glycopeptides. Future work will examine the frequency of occurrence of these factors and their dependence on glycopeptide abundance.

In terms of retention time monitoring for features 1 and 10 listed in the preceding paragraph, the length of permissible retention time depends on the elution length of glycopeptide groups reported by identification software. For sialylated glycans, this length is calculated as 4X higher than the computed median width of the sialylation group (S1-S4) and is within the spectral searching software’s reported envelope of their XIC. Both HCD and IT spectra are examined, and both must not meet criteria above for a peak to be flagged (marked red and annotated).


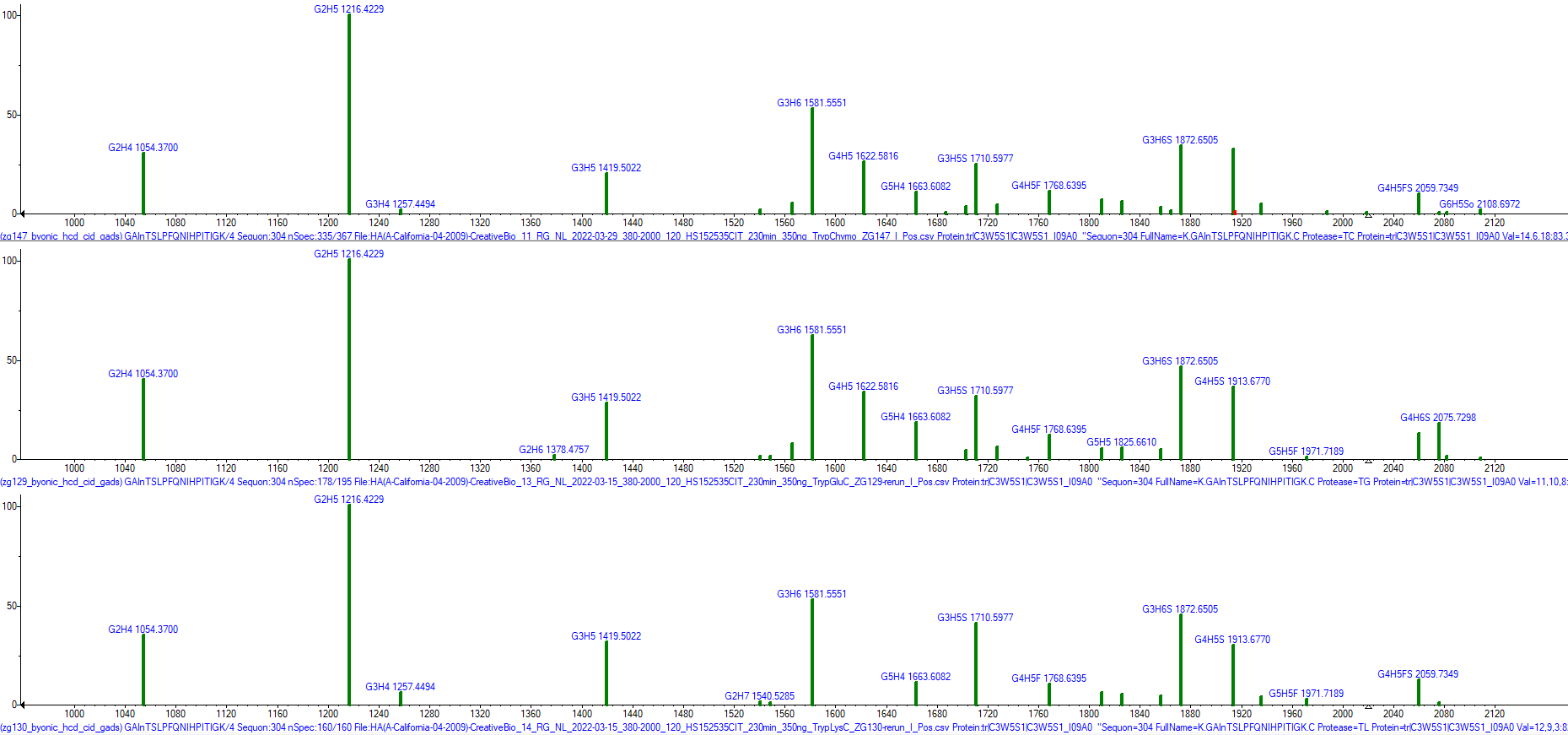


Figure S11. **GADS comparison of different proteases with variation in G4 complex peaks**

GADS compared are from protein HA-CA09 at glycosylation site 304.

Table S3. **List of raw files and their corresponding number of total glycopeptides using Byonic software**

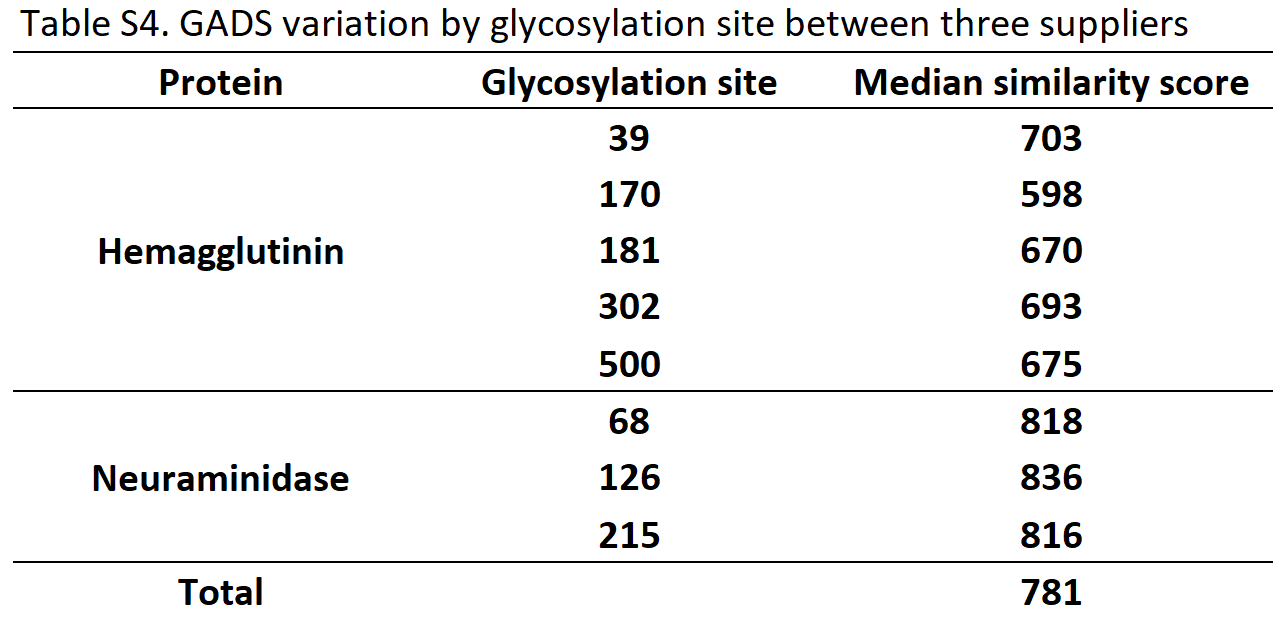


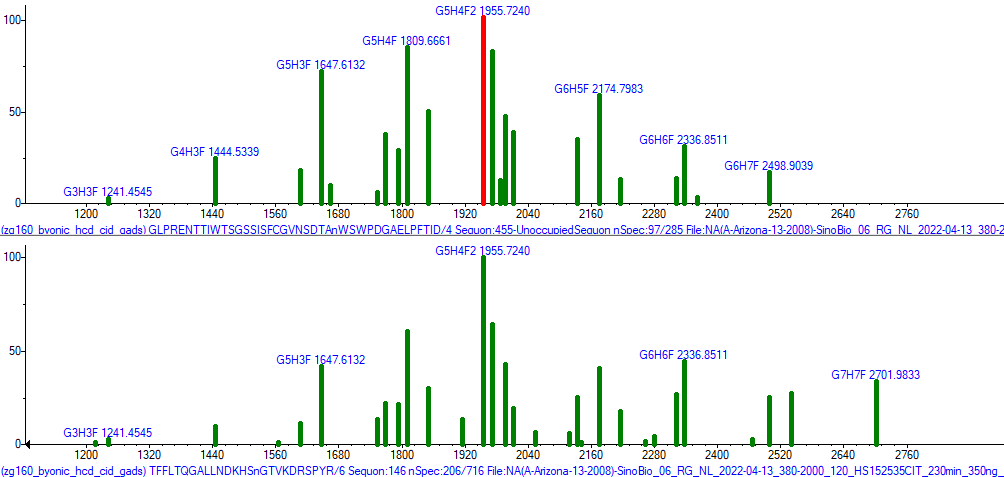
Figure S12. GADS comparison of inter-strain glycosylation sites 146 and 455 in protein NA-AZ08.


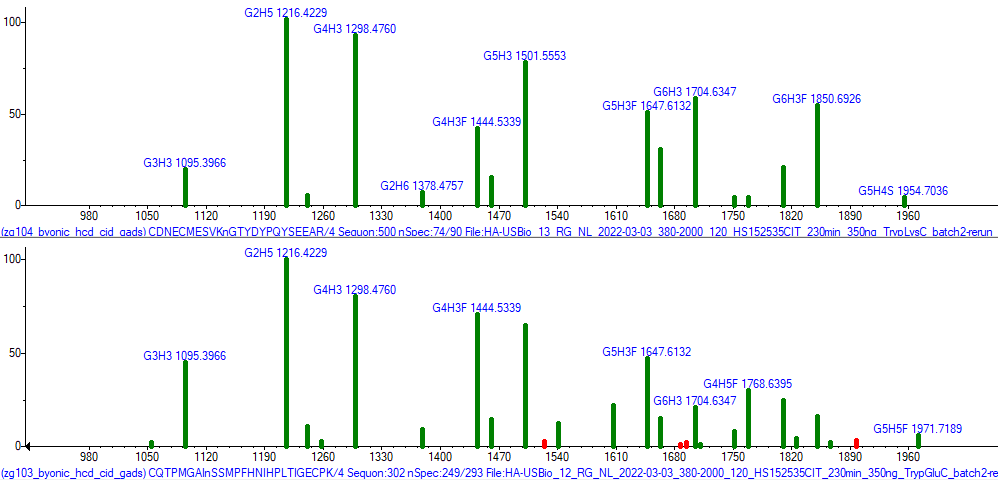


Figure S13. GADS comparison of inter-strain glycosylation sites 302 and 500 in protein HA-HK97


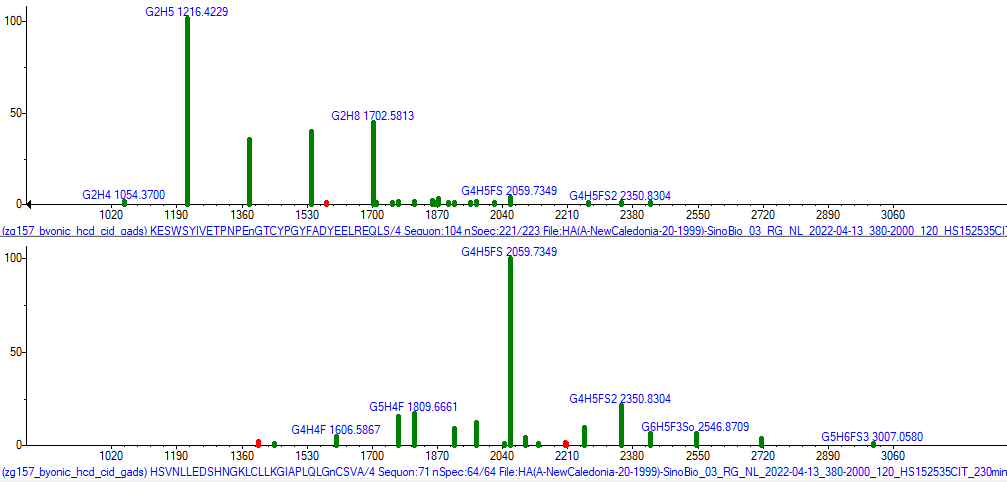


Figure S14. GADS comparison of inter-strain glycosylation sites 71 and 104 in protein HA-NC99


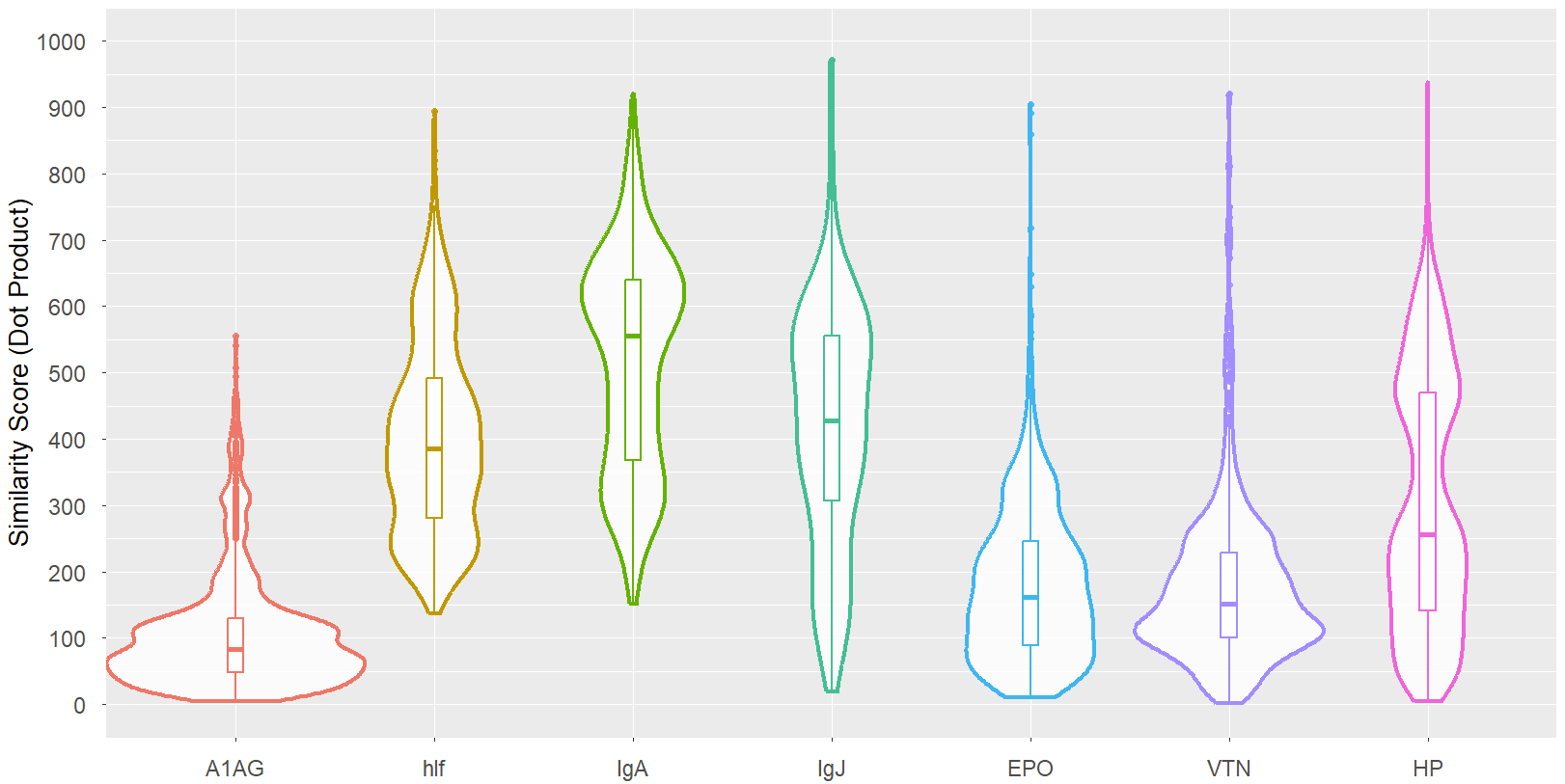


Figure S15. **Violin plot comparing glycan distributions of HA with unrelated proteins**

Page S-1

To illustrate application of GADS similarity scores for entirely different sequence, this shows variations in GADS. Similarity scores between GADS were compared between HA and unrelated proteins such as A1AG, native lactoferrin (hlf), Immunoglobulin A (IgA), Immunoglobulin J (IgJ), Erythropoietin (EPO), Vitronectin (VTN), Haptoglobin (HP). Y-axis represents similarity as calculated using dot product. Box and whiskers plots are included within each violin plot show the four quartiles of the data distribution.


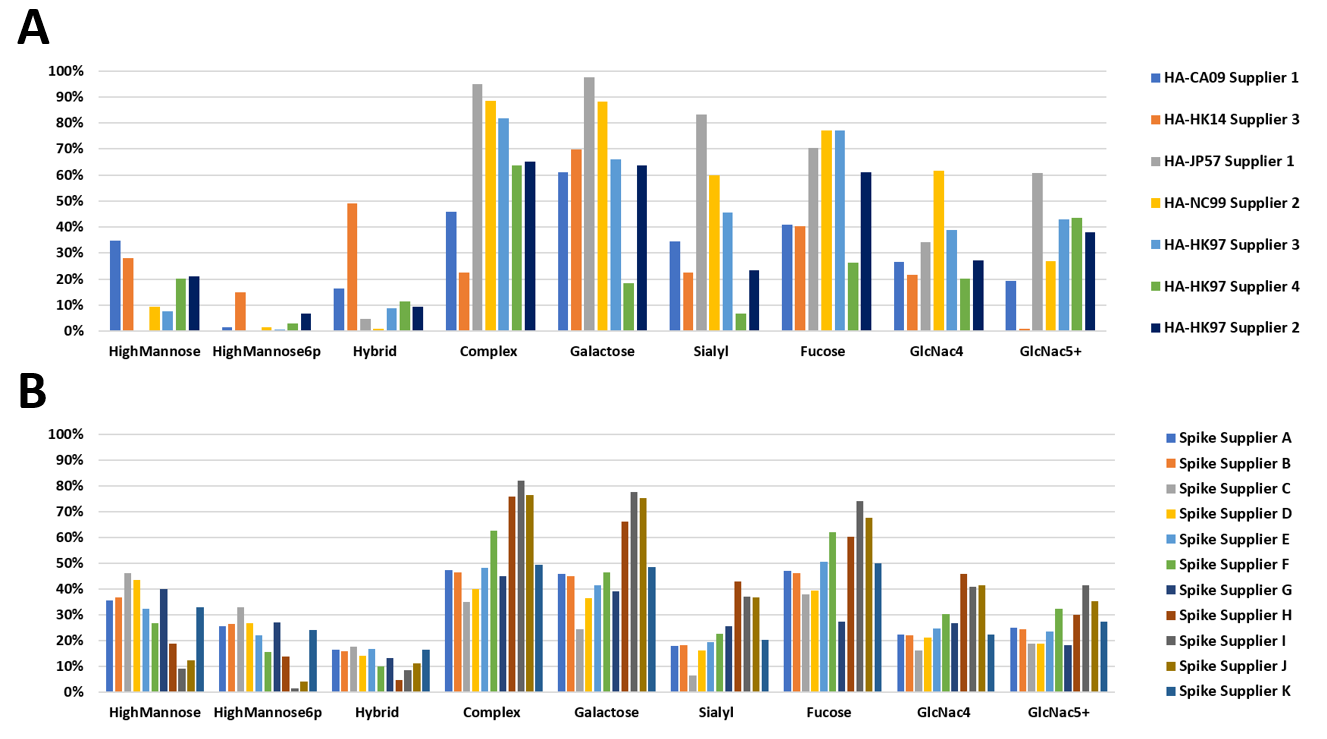


Figure S16. **Comparison of N-glycan types between hemagglutinin and SARS-CoV-2 spike protein**

Abundances of glycan types across all sites and were compared for A) hemagglutinin and B) Sars-CoV-2 spike protein. Different color bars represent different sources of protein.
